# Visualizing translocation dynamics and nascent transcript errors in paused RNA polymerases *in vivo*

**DOI:** 10.1186/s13059-015-0666-5

**Published:** 2015-05-15

**Authors:** Masahiko Imashimizu, Hiroki Takahashi, Taku Oshima, Carl McIntosh, Mikhail Bubunenko, Donald L. Court, Mikhail Kashlev

**Affiliations:** Center for Cancer Research, National Cancer Institute, Frederick, MD 21702 USA; Medical Mycology Research Center, Chiba University, 1-8-1 Inohana, Chuo-ku, Chiba 260-8673 Japan; Graduate School of Biological Sciences, Nara Institute of Science and Technology, 8916-5, Ikoma, Nara 630-0192 Japan

## Abstract

**Background:**

Transcription elongation is frequently interrupted by pausing signals in DNA, with downstream effects on gene expression. Transcription errors also induce prolonged pausing, which can lead to a destabilized genome by interfering with DNA replication. Mechanisms of pausing associated with translocation blocks and misincorporation have been characterized *in vitro*, but not *in vivo*.

**Results:**

We investigate the pausing pattern of RNA polymerase (RNAP) in *Escherichia coli* by a novel approach, combining native elongating transcript sequencing (NET-seq) with RNase footprinting of the transcripts (RNET-seq). We reveal that the G-dC base pair at the 5′ end of the RNA-DNA hybrid interferes with RNAP translocation. The distance between the 5′ G-dC base pair and the 3′ end of RNA fluctuates over a three-nucleotide width. Thus, the G-dC base pair can induce pausing in post-translocated, pre-translocated, and backtracked states of RNAP. Additionally, a CpG sequence of the template DNA strand spanning the active site of RNAP inhibits elongation and induces G-to-A errors, which leads to backtracking of RNAP. Gre factors efficiently proofread the errors and rescue the backtracked complexes. We also find that pausing events are enriched in the 5′ untranslated region and antisense transcription of mRNA genes and are reduced in rRNA genes.

**Conclusions:**

In *E. coli*, robust transcriptional pausing involves RNAP interaction with G-dC at the upstream end of the RNA-DNA hybrid, which interferes with translocation. CpG DNA sequences induce transcriptional pausing and G-to-A errors.

**Electronic supplementary material:**

The online version of this article (doi:10.1186/s13059-015-0666-5) contains supplementary material, which is available to authorized users.

## Background

RNA polymerase (RNAP) transcribes DNA of different structural and chemical sequences. Interaction of RNAP with some of these sequences results in transcriptional pausing, which occurs on average every 100 bp of transcribed DNA *in vitro* [1]. Regulation of elongation via pausing has a variety of physiological consequences [1]. In prokaryotes, the RNAP pausing/anti-pausing system that utilizes RfaH protein controls expression of genes involved in DNA transfer and virulence [2, 3]. Many regulatory events derived from pausing appear to be localized in promoter-proximal regions in eukaryotes or the 5′ untranslated region (UTR) of mRNA genes in prokaryotes [2, 4–6]. For example, eukaryotic RNAPII tends to pause in a region located ≤100 bp downstream of a transcription start site, and is controlled by accessory protein factors such as NELF/DSIF [4, 7]. These paused polymerases allow a rapid transcription response to environmental stimuli and are used during development in higher eukaryotes [4, 6]. The RNAPII pausing at promoter-proximal regions in eukaryotes also plays a critical role in protecting these regions from adopting repressive chromatin structures, thereby maintaining an open promoter complex for highly expressed genes [8, 9]. In prokaryotes, pausing plays a key role in transcription attenuation and termination and in synchronization of transcription and translation [1, 3, 10].

An elongation complex (EC) consists of RNAP bound to double-stranded DNA and the RNA-DNA hybrid with the 3′ end of the RNA positioned in the active center of the enzyme [11]. The hybrid length fluctuates between 9-bp and 10-bp length depending on the translocation state of RNAP. After phosphodiester bond formation, the movement of the RNA-DNA hybrid back along the catalytic cleft vacates the active center, enables binding of the next NTP and reduces the length of the RNA-DNA hybrid from 10 to 9 bp in a process called translocation [1]. Translocation is a smooth process except in cases where certain DNA sequences impose an intrinsic translocation barrier [1, 12]. This block of translocation as well as the inhibition of the bond formation after translocation causes RNAP pausing [1]. Protein factors exist that strengthen or weaken pausing by targeting translocation, such as the archaeal/eukaryotic Spt5 and bacterial NusG/NusA [3, 13, 14] as well as the Nun/N transcription termination/antitermination proteins of lambdoid phages [1, 15]. Pausing of EC within the post-translocated or pre-translocated state is enhanced when an RNA hairpin is formed immediately upstream of the hybrid [16, 17].

Some pausing signals in *Escherichia coli*, such as *ops* sequence, involve backtracking of RNAP along DNA [18]. Backtracking stabilizes pausing [12, 19] and leads to extrusion of one or more nucleotides of the 3′ RNA end beyond the active center [20]. A stably backtracked EC forms a roadblock to DNA replication [21], which can be highly toxic to the cell [22–24]. A direct assessment of transcription fidelity by RNA-seq *in vivo* and *in vitro* showed that an error at the 3′ end of a nascent RNA causes long transcription pausing by inducing RNAP backtracking [25]. It was also shown that transcription errors cause some heritable phenotypic changes [26, 27], which have been thought to affect aging [28] and carcinogenesis [29, 30]. Bacterial GreA and GreB or eukaryotic TFIIS proteins induce endonucleolytic RNA cleavage of any extruded 3′ RNA, with or without errors, thereby allowing renewed transcription in the backtracked EC [31, 32], which ensures better fidelity and removes the DNA replication barrier [22–25].

Extensive biochemical and single-molecule experiments have identified the steps involved in pausing *in vitro* [1]: Pausing can be caused by (i) a misalignment of incoming NTP and complementary template DNA base within the active site of the post-translocated RNAP [33], and (ii) an intrinsic barrier caused by DNA sequence during forward translocation from the pre-translocated state [13, 34]. This latter type of pausing can be stabilized by backtracking [12]. However, little is known about how broadly these mechanisms for pausing identified *in vitro* are involved in transcription regulation *in vivo*.

In the present work, we employed native elongating transcript sequencing (NET-seq) [35] to identify RNAP pause sites and error hotspots in the *E. coli* chromosome by making an assumption that transcription errors contribute to pausing *in vivo*. After paused RNAP complexes are isolated from the genome, RNases are used to trim excess RNA from the 5′ ends leaving only the nascent RNA that is protected by RNAP. Thus, RNET-seq stands for RNase footprinting followed by NET-seq. A previous *in vitro* study showed that an RNAP forming an EC protects different lengths of the 3′-proximal transcript from trimming by RNases A and T1 depending on the EC translocation state [36]. Post-translocated, pre-translocated, and backtracked complexes protect 14-nucleotide (nt), 15-nt and >15-nt segments of the RNA, respectively [36]. Importantly, because the very 3′ end of the RNA is extruded to a narrow pore from the active center of the enzyme during backtracking, the extruded RNA remains inaccessible to RNases increasing in length as backtracking increases [36]. Thus, paused RNAP in either the pre- and post-translocated states as well as at different backtracked distances were monitored over the entire genome. The unique properties of our RNET-seq approach provided an opportunity to dissect the core mechanisms of different types of pausing in living cells.

## Results

### Gre factors reduce pausing in the 5′ UTR genome-wide

**Fig. 1:**
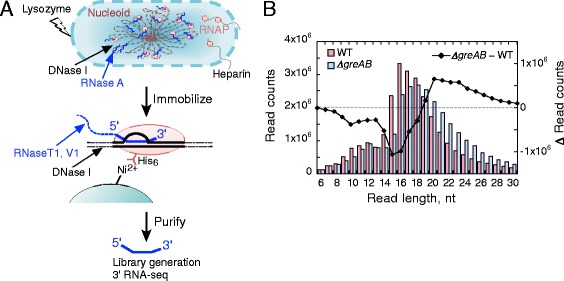
RNET-seq, the read-length-specific NET-seq approach for the analysis of transcriptional pausing and errors *in vivo*. **(a)** An overview of the preparation of the RNA samples for RNET-seq. The 3′ RNA transcripts protected by RNAP from RNases were isolated from *E. coli* cells. **(b)** Different distribution of read lengths between the WT and *ΔgreAB* cells

We employed RNET-seq on the wild-type (WT) *E. coli* strain and an isogenic strain deficient in genes for GreA and GreB (*ΔgreAB*). Gre factors and their eukaryotic analog TFIIS rescue backtracked complexes of RNAP [1]. Briefly, the cells were rapidly lysed via spheroplasting, and the transcribing RNAPs were released from the genomic DNA by digestion with DNase I (Fig. 1A). Any ribosomes involved in co-transcriptional translation were separated from RNAP by digestion with RNase A. During the cell lysis heparin was present to inhibit nonspecific binding of RNAPs to DNA and RNA [37]. All RNAPs, including those associated with the fragmented double-stranded DNAs and their 5′-truncated nascent RNAs, were immobilized on Ni^2+^-NTA beads through the hexa-histidine-tagged β’ subunit [38] and then extensively washed with a high-salt buffer (see “Materials and methods”). The purification was done in the native conditions not involving DNA-protein crosslinking. The 5′ ends of the transcripts in ECs were trimmed with RNase T1/V1 (V1 digests double-stranded RNAs in nascent transcripts, which are resistant to T1) to leave a minimal length of RNA protected by RNAP (Fig. 1A). The RNases were subsequently removed by further washing of the beads. Next, elution with imidazole generated ECs carrying ~6- to 30-nt long transcripts (Fig. 1B; Fig. S1A in Additional file 1). The predominant RNA length distribution was consistent with nucleotide lengths of nascent RNA protected by RNAP from *in vitro* digestion by different RNases in active and backtracked ECs (Fig. 1B; ≥14 nt) [36]. The imidazole eluate also contained shorter <14-nt RNA species (Fig. 1B; Fig. S1A in Additional file 1), which preferentially mapped to the transcription start site regions of the *E. coli* genome (Fig. S2 in Additional file 1), indicating that these short reads derived from active transcription initiation complexes and/or moribund abortive initiation complexes [39]. The nascent RNAs isolated from the *ΔgreAB* strain were longer than those from the WT strain (Fig. 1B) and peaked at 18 nt versus 16 nt, suggesting an enrichment of backtracked ECs, which is expected to occur in the absence of Gre-dependent 3′ RNA cleavage.

**Fig. 2:**
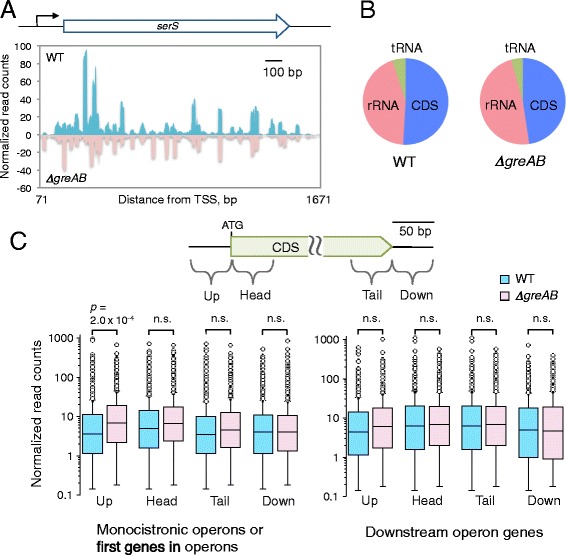
Comparison of genome-wide transcription in *E. coli* WT and *ΔgreAB* cells. **(a)** A transcription pausing profile of the *serR* gene. *TSS* transcription start site. **(b)** Mapped sequencing reads from paused RNAP complexes carrying mRNA (coding DNA sequence (*CDS*)), tRNA and rRNA. **(c)** GreAB proteins reduce pausing in 5′ UTRs of *E. coli* mRNA genes. Each box plot represents the quartile of normalized read counts in a 50-bp window for each gene body: upstream (*Up*), head, tail, and downstream (*Down*). mRNA genes with normalized read counts >0.1 (*n* = 1847 for left panel and *n* = 882 for right panel) were used for the analysis. The *p*-value of two-tailed *t*-test is shown for a pair with statistically significant difference between the WT and *ΔgreAB* data. The *p*-values >0.05 are labeled as non-significant (n.s.)

We investigated the genome-wide landscape of RNAP pausing by using high quality ≥21-nt sequencing reads (Fig. S3 in Additional file 1) for a subset of the 5′-trimmed nascent transcripts isolated from WT and *ΔgreAB* cells. Using the ≥21-nt reads allowed an unambiguous mapping of these reads to the *E. coli* genome compared with their shorter counterparts (see “Materials and methods”). We detected pausing patterns in *E. coli* genes that are consistent with a previous ChIP-chip analysis [40]; for instance, both methods detected increased RNAP pausing in the promoter-proximal region of the *serS* gene for seryl-tRNA synthetase (Fig. 2A) [40]. Approximately 50 % of all pause sites in WT and *ΔgreAB* cells mapped to mRNA genes versus the rRNA and tRNA genes (Fig. 2B). This number is in sharp contrast to an RNA-seq analysis using total cellular RNA, where <2 % of the reads in *E. coli* mapped to mRNA genes while the rest mapped to rRNA and tRNA genes [41]. The dramatic depletion of the rRNA and tRNA transcripts in our RNET-seq data argues that mRNA transcription is much more susceptible to pausing than rRNA and tRNA transcription. This is consistent with the findings that (i) increased density of transcribing RNAPs leads to suppression of backtracking of the leading RNAP by the trailing one [42] and (ii) Nus protein complexes are required for transcription of operons containing rRNA and tRNA genes since these complexes inhibit pausing [43, 44].

It has been shown that Gre factors affect transcription initiation, elongation and fidelity [25, 31, 45–48]. However, a role for Gre factors in the global control of transcription pausing *in vivo* remains poorly understood. We addressed the impact of Gre proteins on pausing using RNET-seq analyses, comparing the nascent transcript levels in the WT and *ΔgreAB* cells for each mRNA gene by calculating the number of normalized reads per gene (Fig. S4A in Additional file 1). The value is affected by three parameters: (i) promoter strength, (ii) frequency of pausing, and (iii) duration of pausing during elongation [35]. Note that because the nascent transcript levels were calculated for ≥21-nt reads, paused polymerases within the initiation region (corresponding to <14-nt reads) were not included. We observed a weaker correlation between strains with and without Gre for mRNA reads (*r* = 0.75) compared with rRNA (*r* = 0.99) or tRNA reads (*r* = 0.89) (Fig. S4 in Additional file 1).

To determine if transcription of specific segments of mRNA genes and operons is targeted by Gre factors, we calculated the number of normalized reads for all mRNA genes by dividing them into four separate regions: 50 bases immediately upstream of the start codon (Up); 50 bases just downstream of the start codon (Head); 50 bases just upstream of the stop codon (Tail); and 50 bases just downstream of the stop codon (Down) (Fig. 2C). There was only one significant difference with and without Gre present, and it was found in the Up region of monocistronic operons as well as in the first genes in polycistronic operons (Fig. 2C). Because the median length of 5′ UTRs in *E. coli* is 36 bases [49], we suggest that Gre factors suppress RNAP backtrack pausing in the 5′ UTRs without having a significant effect on the distal parts of genes and operons. Although we arbitrarily selected 50 bases for the Up region regardless of any variability in the 5′ UTR lengths, we observed stronger suppression of pausing by Gre proteins in Up regions that contained 5′ UTRs with lengths of around 50 bases (Fig. S4B in Additional file 1). This finding is consistent with the proposed role of co-transcriptional translation in suppression of backtrack pauses in *E. coli* [50].

### RNET-seq using ≥21-nt reads identifies robust pausing signals and their non-random distribution

**Fig. 3:**
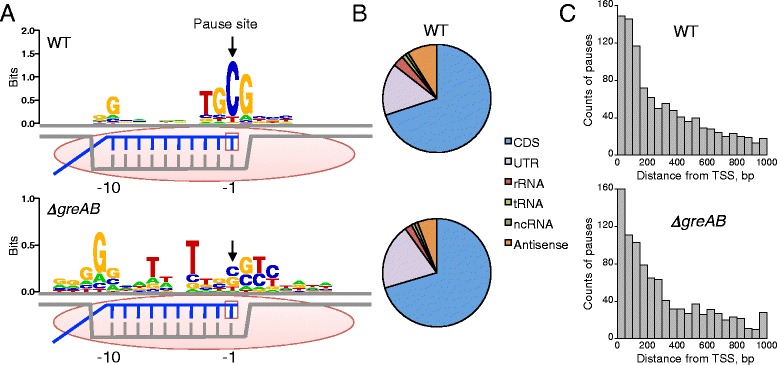
Transcription pausing detected by RNET-seq in *E. coli* WT and *ΔgreAB* cells. **(a)** Pause-inducing elements (PIEs) of the non-template DNA strand. Information content is represented by sequence logos [51]. Positions −1 and −10 of DNA (*gray*) correspond to the RNA (*blue*) 3′ and 5′ ends of the RNA-DNA hybrid within RNAP (*pink oval*) in pre-translocated ECs. The active site is shown as an *empty square. P*(0.9, 100) and *P*(0.9, 160) were used for WT (*n* = 758) and *ΔgreAB* (*n* =419), respectively (see main text for the parameters). A frequency matrix and MAP scores for the PIEs are shown in Table S1 in Additional file 2. **(b)** Categorization of all RNAP pauses by RNA type. The non-coding RNA (*ncRNA*) and antisense RNA were defined using the gene annotation file of *E. coli* (see “Materials and methods”). **(c)** Pausing frequently occurs in regions proximal to transcript start sites (*TSS*). For panels B and C, *P*(0.7, 100) is used in order to increase the number of samples. Note that even when using this reduced stringency the consensus sequence for pausing remains unaffected (Fig. S6B in Additional file 1)

We examined every base pair position throughout the genome in both orientations as a potential pause site by determining the number (or depth) of reads (*δ*) for each mapped genomic position as well as the fraction *ϕ*) of those reads in which that position is at the 3′ RNA end. To identify robust pause sites by RNET-seq, we selected only those genomic positions where RNAP paused with a frequency *ϕ* that is arbitrarily high (≥0.9; Fig. S5 in Additional file 1). Thus, *P*(*ϕ*, *δ*), where *ϕ* is the minimal fraction of having 3′ RNA ends in the mapped reads and *δ* is the minimal read depth for any genomic position. We chose *δ* to be 100 for WT and 160 for *ΔgreAB*, which normalized these respective numbers for each strain since there were 1.6-fold more total reads in the *ΔgreAB* strain. The high *ϕ* parameter allowed us to define a reliable pause-inducing element (PIE) for WT or *ΔgreAB* cells (Fig. 3A). The PIEs identified in both strains were different from each other and had high scores for information content, which were similar to other already-known transcription factor motifs in *E. coli* (Fig. 3A; Tables S1 and S2 in Additional file 2; Method S1 in Additional file 3 for calculation) [51–53].

The PIE for the WT strain consisted of the upstream and downstream subelements: (i) a G_−10_ located at the upstream edge of the 10-bp RNA-DNA hybrid in a pre-translocated EC [54]; and (ii) a TGC_−1_G_+1_ sequence spanning the RNAP active center, where the −1 DNA base of the non-template strand corresponds to the 3′ RNA base in a paused EC. Notably, the C_−1_G_+1_ sequence accounts for about half of the total score for information content in the motif (Table S1 in Additional file 2). The PIE derived from the *ΔgreAB* data consisted of G_−11_ and T_−4_ T_−7_ (Fig. 3A). Excluding from the *ΔgreAB* data those pause sites shared by WT and *ΔgreAB* cells did not significantly change the *ΔgreAB* PIE (Fig. S6A in Additional file 1). These data argue that major pause sites found in the WT and the *ΔgreAB* cells are very different *in vivo*, contrary to an *E. coli* NET-seq study that made a similar comparison [55]. Our previous studies showed that the weak U-dA base pairs in the RNA-DNA hybrid in bacterial and eukaryotic ECs stimulate RNAP backtracking *in vitro* [25, 56]. The similarity between *in vitro* and *in vivo* data indicates that lack of Gre proteins in the cell enriched the backtracked RNAP. This enrichment predominantly occurs at the T-rich signal (T_−4_ T_−7_ motif) coding for the unstable U-dA base pairs in the EC [12]. The two distinct PIEs observed for WT and *ΔgreAB* cells were also found when using less stringent *ϕ* and *δ* values to define pausing (Fig. S6B in Additional file 1). 95 % (WT) and 94 % (*ΔgreAB*) of the pause sites identified using these parameters showed high uniqueness for the read mapping (Fig. S7 in Additional file 1) [57]. Other pause sites with lower mapping uniqueness were mainly located in the multi-copy rRNA genes or in the multi-copy insertion sequence (IS) gene coding for transposase (Fig. S7 in Additional file 1; see “Materials and methods”).

Among all types of genes, pausing was primarily detected in mRNA genes in both WT and *ΔgreAB* cells (Fig. 3B). Considering that the UTRs are ~10–20 times shorter than the coding DNA sequences (CDSs) in *E. coli* mRNA, pausing density in the UTRs was found to be higher than in the CDSs. Figure 2C shows an increased frequency or duration of pausing in 5′ UTRs (50 bp) in *ΔgreAB* compared with WT cells, and Fig. 3C shows no significant difference in the frequencies of those pauses within the 200 bp downstream of transcription start sites. Thus, we suggest that the duration of those pauses was increased due to RNAP backtracking in 5′ UTRs of *ΔgreAB* cells. This notion is supported by the *ΔgreAB* PIE specifically having the backtracking T_−4_ T_−7_ signal coding for a weak RNA-DNA hybrid (Fig. 3A; Fig. S6A in Additional file 1) [12].

### Putative role of antisense transcription in gene repression

**Fig. 4:**
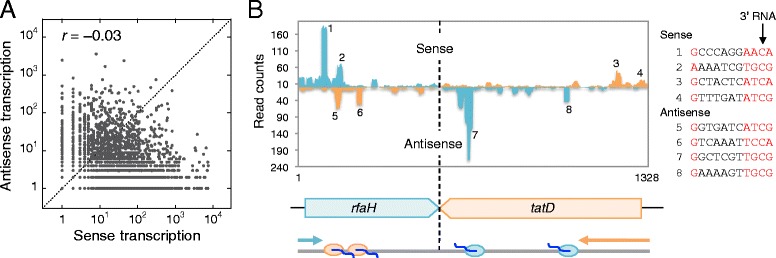
RNAP pauses during antisense transcription detected by RNET-seq. **(a)** Scatter plot of antisense and sense transcription in the WT strain. Each dot represents a 50-bp “tail” (Fig. 2C) region of a gene. The Pearson’s correlation coefficient (*r*) is shown, and y = x (*dotted line*) represents a positive correlation. **(b)** Convergent transcription and pauses in the *rfaH* and *tatD* genes of WT cells. The reads mapped to plus and minus strands of genomic DNA are shown in *blue* and *orange*, respectively. The sequences for pause sites (labeled by *1–8*) are shown

Antisense or convergent transcription has been found in all kingdoms of life [58–61]; however, its physiological role remains obscure. Our RNET-seq analysis revealed that polymerase often transcribed in both directions through the same regions. In some cases, two convergent genes were involved, and in others, it appeared that antisense transcription occurred in an annotated non-coding sequence. Pausing during antisense transcription was the third most common type out of all pausing events in *E. coli* (Fig. 3B). A scatter plot of the normalized read counts obtained in the sense and antisense directions for the 50-bp Tail region showed a slightly negative correlation between antisense and sense transcription (Fig. 4A; the ratios of antisense transcript levels to sense levels were plotted largely across y = x, representing positive correlation). This pattern suggests that converging RNAPs interfered with each other. A similar trend was observed in the 50-bp Up and Head regions in genes (Fig. S8A in Additional file 1), indicating a robust interference effect wherever convergent transcription occurred. For example, the *rfaH* and *tatD* genes, which are located in a head-to-head orientation on the chromosome, show the same pausing pattern (Fig. 4B). These two genes are expressed at low levels in exponentially growing cells under the conditions we employed for RNET-seq [62, 63]. Notably, *rfaH* showed a strong cluster of pauses near the 5′ end of the gene (sites 1 and 2), which coincided with strong pauses in the antisense direction for the *tatD* gene (sites 5 and 6). A similar cluster of pauses was observed in *tatD* where progression of RNAP appeared to be confined to the 5′ part of the *tatD* gene (sites 3 and 4), which we interpreted as a sign of collision between the sense transcription and the antisense traffic streaming from *rfaH* (sites 7 and 8). Interestingly, RfaH protein is an anti-pausing transcription factor predominantly expressed in stationary growth phase [3]. This implies a potential effect of antisense pausing on the suppression of gene expression under control of the RfaH protein in the early growth phase. We noted that all pause sites for convergent transcription in *rfaH*/*tatD* were similar to other G_-10…_C_−1_G_+1_ PIEs where unidirectional transcription occurred (Fig. 4B). A similar antisense pattern was observed in the *insB* gene coding for the transposase of the IS*1* mobile element (Fig. S8B in Additional file 1) [64]. These pauses at IS*1* in WT cells represented ~2 % of all pauses identified with parameter values *P*(0.9, 100). We propose that the antisense pausing in IS*1* may be essential to prevent spontaneous bursts of transposase production that destabilize *E. coli* genome.

### The G-dC base pair at the 5′ end of the RNA-DNA hybrid interferes with translocation

*E. coli* RNAP strongly protects the 14-nt segment of the nascent transcript from degradation by RNase T1 and RNase A in an active post-translocated EC *in vitro* [36]. Likewise, in the pre-translocated state, RNAP protects the 15-nt transcript, whereas backtracking increases the protection to ≥16 nt depending on the backtracking distance [36]. Therefore, each of these different length reads in the RNET-seq contains information about translocation and backtracking states of RNAPs associated with them during elongation and pausing *in vivo*. We employed this useful information to analyze the dynamics of RNAP translocation and backtracking associated with pausing *in vivo*.

**Fig. 5:**
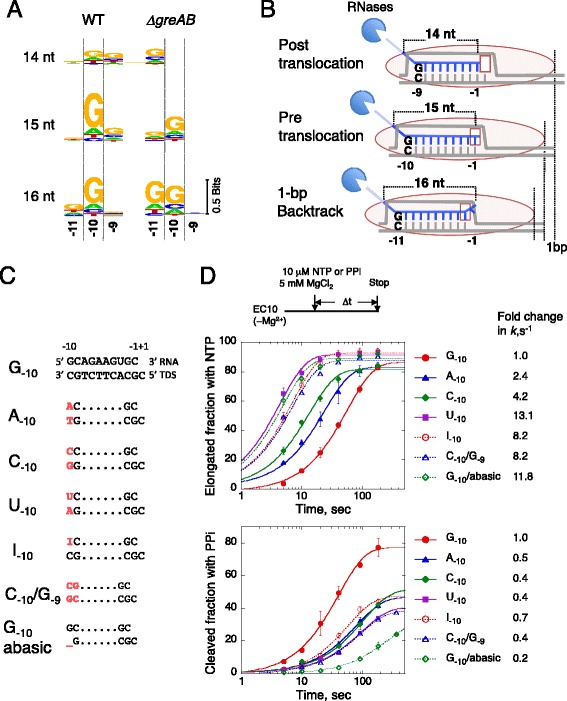
The G-dC base pair at the 5′ end of the RNA-DNA hybrid interferes with RNAP translocation *in vivo* and *in vitro*. **(a)** PIEs generated by the single-length RNET-seq analysis for 14-, 15- or 16-nt reads from WT and *ΔgreAB* cells. DNA positions −9, −10 and −11, where −1 corresponds to the 3′ RNA base, are shown. Pausing was defined by *P*(0.9, 50). The full-length PIEs are shown in Figs. S9 and S10 in Additional file 1. For 14-nt reads, the pause sties of mapq_mean_ >10 are used (*n* = 286 and 258 for WT and *ΔgreAB*, respectively; Fig. S11 in Additional file 1). **(b)** Model for robust transcription pausing in the post- (14 nt), pre-translocated (15 nt) or 1-bp backtracked (16 nt) state according to the −9, −10 or −11 position of the riboG-dC. **(c)** Ten-nucleotide RNA strands (top) and the template DNA strands (*TDS*) in the ECs used for the biochemical assay. The RNA and template DNA bases, carrying sequence different from the original G_−10_ scaffold, are indicated in *red*. **(d)** Effects of different −10 and +1 bases on the elongation (upper) and pyrophosphorolysis (lower) of an EC carrying a 10-nt transcript (EC10). Reaction scheme is shown at the top. The apparent rate constants (*k*) for these two reactions were obtained by fitting the data to single-exponential curves. The mean values of two or three independent experiments ± standard deviations are shown. *PPi* pyrophosphate

We compared the individual PIEs for each separate length of RNA read from 14 upto 23 nt across the entire *E. coli* genome. This single-length RNET-seq analysis revealed informative differences in distance between the upstream and downstream subelements of the PIEs, which were dependent on the specific read length and corresponding translocation state of RNAP. The observed differences also depended on the presence or absence of Gre factors (Fig. 5A; Figs. S9 and S10 in Additional file 1). In the post-translocated pauses containing 14-nt long reads in WT cells, the G_−10_ signal of the upstream PIE identified using bulk reads ≥21 nt in length was significantly reduced and G_−9_ was moderately favored (compare Figs. 3A and 5A). G_−9_ was more prominent in positions with low mapping quality (Fig. S11 in Additional file 1), which we did not investigate further. In WT cells, G_−10_ was the most reliable upstream PIE signal in the pre-translocated state (15-nt long reads) or the 1-bp backtracked state (16-nt long reads) (Fig. 5A). A similar trend was observed in *ΔgreAB* cells except that G_−11_ was the most favored for the ≥1-bp backtracked states (Fig. 5A; Fig. S10 in Additional file 1).

The post-translocated EC of bacterial RNAP has been shown to contain a 9-bp RNA-DNA hybrid [54], which also implies that the pre-translocated and 1-bp backtracked ECs contain a 10-bp hybrid. In the 1-bp backtracked EC, the 3′ nucleotide in the RNA was extruded into the secondary channel in RNAP. Thus, the riboG-dC base pair in the −9, −10 or −11 position relative to the 3′ RNA end corresponds to the upstream end of the RNA-DNA hybrid in post-translocated, pre-translocated and 1-bp backtracked states, respectively (Fig. 5B). This riboG-dC base pair at the upstream end of the hybrid appears to be very important for pausing to occur in any one of the three translocation registers of RNAP (Fig. 5B). We interpreted that this base pair prevents rapid exchange between any of these states. The favored G_−10_ in the WT PIE also suggests that Gre-mediated RNA cleavage rapidly rescues backtracked ECs and allows RNA to be elongated back to the pre-translocated pause in WT cells (see “Discussion” for the detailed mechanism).

To validate a role for a riboG-dC base pair at the upstream end of the RNA-DNA hybrid in pausing, we performed *in vitro* assembly of ECs with RNAP purified from the *ΔgreAB* strain and synthetic DNA-RNA hybrid scaffolds carrying different bases at the −9 and −10 positions relative to the downstream PIE (TGC_−1_G_+1_; Fig. 5C) [25]. The bias in translocation equilibria of these ECs was analyzed by measuring the rates of RNA extension and pyrophosphorolysis [12]. ECs in the pre-translocated state exhibit a characteristic high rate of pyrophosphorolysis and low rate of RNA extension as opposed to their post-translocated counterparts, which typically exhibit a low rate of pyrophosphorolysis [12]. An EC carrying G_−10_ showed increased pyrophosphorolysis at 10 μM pyrophosphate (PPi), being 1.4-fold more rapid than RNA extension at the same 10 μM concentration of NTP (Fig. 5D). In agreement with the *in vivo* data, this indicates a preferred pre-translocated state for this complex. Substituting G_−10_ for A, C, or U increased the RNA extension and decreased pyrophosphorolysis with A_−10_ causing less dramatic results than C_−10_ or U_−10_ (Fig. 5D). The dramatic effect of a G_−10_ to C_−10_ substitution on translocation, with only a minor effect on thermodynamic stability of the base pairing, argued against a simple view that the more stable RNA-DNA base pairing at the hybrid end interferes with translocation. A G_−10_ to I (inosine)_−10_ substitution in the RNA, which altered the riboG-dC hydrogen bond geometry [65], or replacement of dC_−10_ in template DNA with a non-instructional abasic site (Fig. 5C) also significantly increased the RNA extension rate and reduced pyrophosphorolysis in the complex (Fig. 5D). Thus, we conclude that the particular character of the 5′ riboG-dC base pair of the hybrid was necessary for biasing translocation equilibrium toward the pre-translocated state.

A G_−10_C_−9_ to C_−10_G_−9_ conversion at the upstream end of the hybrid substantially stimulated RNA extension and reduced pyrophosphorolysis in the complex (Fig. 5D). This result indicates that the riboG_−9_-dC base pair inclined translocation equilibrium toward the post-translocated state. Note that the riboG-dC base pair at the −9, −10 or −11 position in the post-translocated, pre-translocated, or 1-bp backtracked state, respectively, likely interacts with the same part of RNAP to restrict the EC mobility on DNA in all three transcription registers (Fig. 5B). Taken together, both *in vivo* and *in vitro* data argue that interaction of RNAP protein with the riboG-dC base pair at the upstream end of the hybrid interferes with RNAP translocation irrespective of the translocation register of the enzyme. This interference, together with the effect of the CpG downstream element on translocation and/or catalysis, determines RNAP pausing in each translocated state (discussed below).

### G-to-A error at the 3′ RNA end induces backtrack pausing genome-wide

**Fig. 6:**
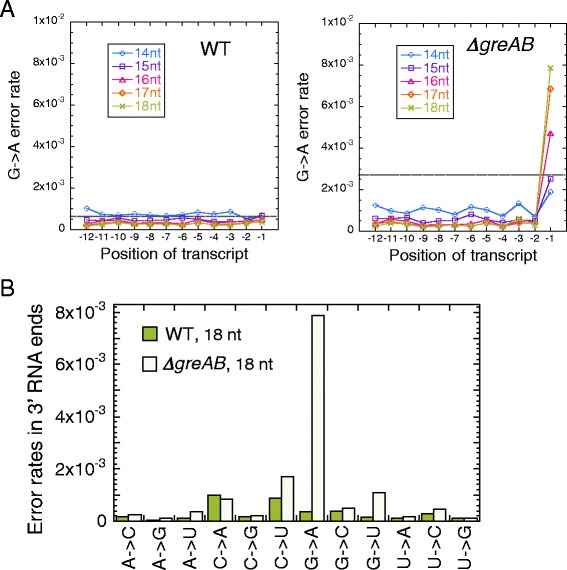
Transcriptional errors detected by single-length (14–18 nt) RNET-seq. **(a)** G-to-A error rates at the 3′ RNA ends are increased in the absence of Gre factors. Position −1 corresponds to the 3′ RNA end. Broken lines represent values for mean error rate + standard deviation in the −12 to −1 positions of the 14- to 18-nt reads. **(b)** Error rates in the 3′ ends of nascent transcripts detected by single-length (18 nt) RNET-seq

It has been reported that a transcriptional G-to-A error in *E. coli* induces prolonged backtrack pausing of RNAP, which can be the most frequent error in the absence of Gre factors [25]. We used this knowledge to examine if the RNET-seq analysis could detect G-to-A errors enriched in the nascent 3′ transcripts of the paused complexes. Indeed, G-to-A errors were significantly enriched at the 3′ RNA ends in *ΔgreAB* cells but not in WT cells (Fig. 6A). The G-to-A errors in *ΔgreAB* cells were also dominant among all 12 possible errors types (Fig. 6B). The highest G-to-A error rate (~8 × 10^−3^) was observed at the 3′ ends in the 18-nt reads of *ΔgreAB* cells; these errors constituted ~1 % of all pausing events. Importantly, the G-to-A error rate steadily declined as the read length decreased from 18 to 14 nt, representing a correlation of the error rate and the backtracking distance (Fig. 6A). This pattern indicates that backtracking was essential for RNAP pausing after the misincorporation. In WT cells, efficient proofreading of the 3′ G-to-A errors by Gre factors in the backtracked RNAP appeared to be responsible for their reduction below the RNET-seq detection limit (Fig. 6A). Other types of errors (e.g., C-to-A or C-to-G) were not significantly affected by deletion of Gre factors (Fig. 6B). We interpreted that these errors represent RNET-seq artifacts. Alternatively, these transcription errors could derive from DNA sequences where the 3′ RNA-DNA mismatches did not induce RNAP backtracking to prevent their proofreading by Gre factors.

RNET-seq also showed an enrichment of some types of errors at variable distances from the 3′ RNA end (Fig. S13 in Additional file 1). For instance, G-to-U errors were highly enriched between the −8 and −6 positions, with the peak position depending on the read length of RNET-seq. G-to-C, A-to-C, U-to-C and U-to-G errors were also enriched in the −3 positions in *ΔgreAB* cells and this enrichment was also dependent on the read length. Note that the average artificial error rates in our sequencing method were on the order of 10^−4^ or less and the sequencing data were of high quality (Figs. S3 in Additional file 1) [25]. These unique properties argue that these errors occurred during transcription *in vivo*. More extensive biochemical analysis is warranted to understand how different types of transcriptional errors located within the RNA-DNA hybrid in an elongating RNAP induce pausing *in vivo*.

## Discussion

RNET-seq has identified G_−10_ and TGC_−1_G_+1_ as robust pausing signals in *E. coli* cells that are similar to pausing sites analyzed by biochemical or single-molecule *in vitro* studies for *E. coli* RNAP and yeast/human RNAPII [12, 66, 67]. In human RNAPII, a poly(G) rather than a single G immediately upstream of the RNA-DNA hybrid was shown to induce strong backtrack pausing rescued by TFIIS, the eukaryotic counterpart of Gre factors [66]. Thus, a core sequence-dependent mechanism for RNAP pausing on bare DNA appears to have similar sequence requirements in prokaryotes and eukaryotes.

How does the C_−1_G_+1_ element spanning the active site of RNAP induce robust pausing *in vivo*? We previously suggested that increased flexibility of the dCMP sugar-phosphate backbone of a CpG dinucleotide in the template DNA strand can cause pausing by interfering with proper alignment of the template DNA base with incoming NTP in the post-translocated state [1, 33]. Similar misalignment of the 3′ RNA end with the template base may cause pausing in the pre-translocated state [1, 12, 68]. This dynamic property of CpG in DNA has been identified by a variety of methods [69–71]. A recent deep-sequencing study of transcription in mammalian cells revealed that RNAPII pausing frequently occurred in gene bodies containing CpG repeats [72]. Importantly, CpG methylation reduces flexibility of the sugar moiety of the dC [73, 74], and also reduces pausing of *E. coli* RNAP *in vitro* [75]. Our *in vitro* experiment using a 5-methyl-dC_+1_ residue introduced into the pausing motif moderately increased RNA extension rate and strongly reduced efficiency of pyrophosphorolysis (Fig. S12 in Additional file 1). Thus, the 5-methyl-dC_+1_ in the template DNA of the PIE appeared to shift the translocation equilibrium to the post-translocated (pyrophosphate-resistant) state.

**Fig. 7:**
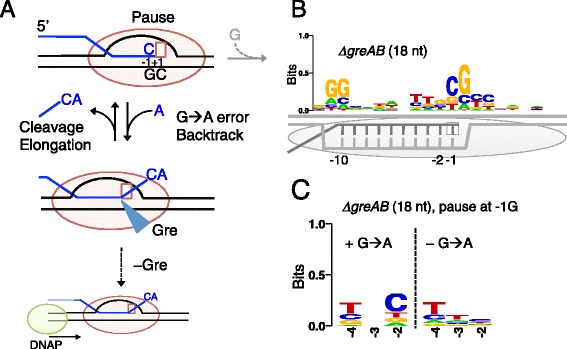
Transcriptional pauses and errors frequently occur at CpG sequences *in vivo*. **(a)** G-to-A error at the 3′ RNA end induces backtracking of RNAP in the C_−1_G_+1_ motif. These pauses are rescued and the errors are corrected by Gre factors. In the absence of Gre factors, the backtracked RNAP imposes a strong barrier to a replicating DNA polymerase (*DNAP*) leading to double-strand DNA breaks [22, 24]. **(b)** The PIE of the non-template DNA in the *ΔgreAB*, which is composed of 1,555 pause sites identified by parameters *P*(0.9, 50). The pre-translocated RNAP (*gray oval*) and the RNA-DNA hybrid are shown. **(c)** The 3′-penultimate C residue in the nascent RNA is favored when a G-to-A error(s) occurs at the 3′ G residue in *ΔgreAB* cells. The two groups for the PIE are shown: the left-side group represents reads containing ≥1 G-to-A errors at the G_−1_ (*n* = 127) and the right-side group represents the reads containing no error at the G_−1_ (*n* = 162). For panels B and C, 18 nt reads were used

**Fig. 8:**
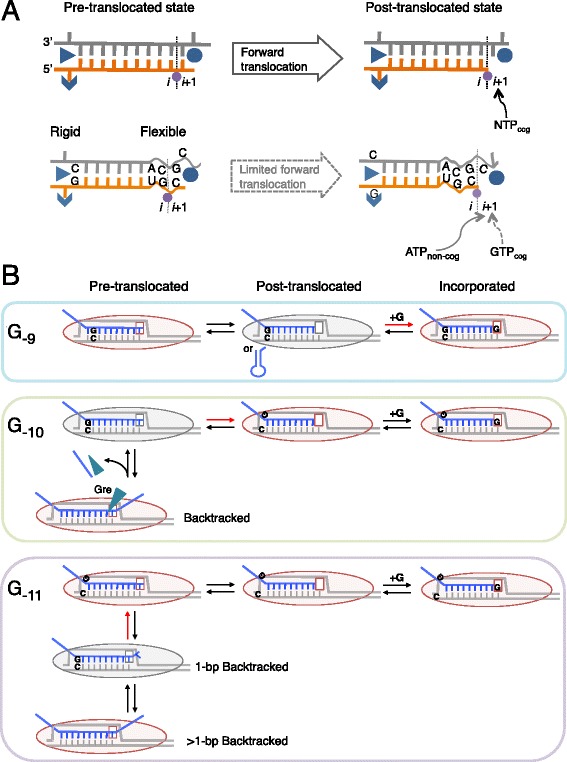
Structural and kinetic models of transcription pausing *in vivo*. (**a**) Structural model. RNAP elongation in a pause-free sequence (*top*) or the PIE (*bottom*) is shown. RNA (*orange*), template DNA strand (*gray*), catalytic Mg^2+^ (*magenta*), and two RNAP domains (*blue*) involved in the 5′ RNA separation from the RNA-DNA hybrid, i.e., Switch 3 (*arrow head*), lid (*triangle*) domain, and the bridge helix of RNAP (*blue circle*) are shown. The 3′ RNA-binding site (*i*) and the NTP binding site (*i* + 1) are also indicated. The 3′ ACGC 5′ sequence in the template DNA and the complementary 5′ UGC 3′ RNA sequence increase the flexibility of their backbones, which decreases cognate GTP (*GTP*_*cog*_) addition and increases non-cognate ATP (*ATP*_*non-cog*_) addition to the 3′ RNA end. The two RNAP domains can interact with riboG-dC at the upstream end of the hybrid, which interferes with the hybrid movement through the catalytic cleft of RNAP. (**b**) Kinetic model. RNAP pauses in the post-translocated (G_−9_ or RNA hairpin, top), pre-translocated (G_−10_, middle), and backtracked states (G_−11_, bottom). RNAPs with the *i* + 1 NTP binding site are shown (*oval shapes* with *empty squares*). Gre factors are indicated by *cyan triangles*. Post- and pre-translocated pauses were mainly observed in WT cells, and backtracked pauses were observed in *ΔgreAB* cells. The rate-determining steps during elongation are indicated by *red arrows*. The RNAP conformations captured by RNET-seq are indicated by *gray ovals*. Note that the GreAB-dependent cleavage, which occurs between *i* and *i* + 1 sites, ultimately converts the backtracked state to the post-translocated state. This state is rapidly converted back to the pre-translocated state prior to the next NTP binding and bond formation at the *i* + 1 site. The presence of activation energy much higher than *k*_B_ *T* in each rate-determining step is assumed for the kinetic description of pausing *in vivo* [1, 39]

Our work shows that G-to-A errors at the 3′ RNA ends cause backtrack pausing of RNAP genome-wide (Fig. 6). Interestingly, this *in vivo* effect was much stronger for G-to-A errors than for any other error types in *E. coli* (Fig. 6B; Fig. S13 in Additional file 1), suggesting a common mechanistic origin of pausing and AMP misincorporation on C_−1_G_+1_ sequence in the non-template strand (G_−1_C_+1_ in the template strand). We propose a mechanism that explains a link between the frequent G-to-A errors and pauses in the PIE (Fig. 7A). An encounter with the G_-10…_C_−1_G_+1_ sequence induces RNAP pausing. During pausing, incorporation of the next cognate GMP decreases while the AMP misincorporation increases, as has been previously shown *in vitro* [25]. Both pausing and misincorporation derive from the flexible sugar backbone of the dC_+1_ template residue (Fig. 8A). The lack of structural constraint interferes with the canonical alignment of GTP with dCMP in the active center and makes the non-canonical alignment of ATP with dCMP more tolerated. Alternatively, the increased flexibility of the dCMP may induce its temporary withdrawal from the active site to stimulate the AMP misincorporation according to the A-rule synthesis recently reported for multi-subunit RNAPs during transcription on abasic DNA sites [76]. The resultant formation of an A-dC mis-pair at the 3′ RNA end induces a prolonged backtracked pause [25], allowing its detection by RNET-seq in *ΔgreAB* cells. Such stable backtrack pauses are major threats to genome stability due to their ability to block DNA replication, which can lead to double strand breaks in DNA (Fig. 7A) [22, 24]. Gre factors stimulate rapid removal of the mis-paired 3′ AMP residue, allowing RNAP to resume elongation at the original PIE (G_-10…_C_−1_G_+1_). We confirmed this model by showing that a C residue in the 3′-penultimate RNA residue was favored in the reads containing the G-to-A error at the 3′ residue, but not in the error-free reads in the PIE derived from backtracked reads (18 nt) in *ΔgreAB* cells (Fig. 7B, C). Thus, our data are fully consistent with the model that both pausing and AMP misincorporation are enhanced at the C_−1_G_+1_ sequence (Fig. 7A).

We showed *in vivo* and validated *in vitro* that the G-dC base pair at the upstream end of the RNA-DNA hybrid interferes with the forward translocation of RNAP (Fig. 5). An X-ray structure of bacterial EC identified lid and Switch-3 domains in RNAP interacting with RNA nucleotides at the upstream edge of the RNA-DNA hybrid (Fig. 8A) [54]. A biochemical study showed that amino acid changes in these domains affected RNAP catalytic activity and translocation [77]. More work is needed to determine the impact of the G-dC base pair at the end of the hybrid on translocation blocking at the PIE. This block may derive from a specific interaction of the G-dC base pair with the RNAP domain(s).

The principal finding of this work is that the location of the G_−10_ nucleotide relative to the TGC_−1_G_+1_ motif in the PIE fluctuates over a 3-bp distance and these fluctuations correlate with the length of the RNET-seq reads (Fig. 5). Because the read length depends on the translocation state of RNAP during pausing *in vivo*, this finding served as a foundation for a kinetic model for the robust pausing mechanism in *E. coli* (Fig. 8B). In paused RNAP, riboG residue at −9, −10, or −11 nt position (G_−9_, G_−10_, or G_−11_) is located at the same distance from RNAP active center in the post-translocated, pre-translocated, or 1-bp backtracked state, respectively (Fig. 8B). The G_−9_, G_−10_, or G_−11_ that pairs with dC in the template at the upstream end of the RNA-DNA hybrid determines the rate of escape from each of these states during transcription. The G_−9_ stabilizes the post-translocated state before backtracking (Fig. 8B). The effect of G_−9_ on pausing is minor compared with that of the TGC_−1_G_+1_ motif, which causes a misalignment of GTP with the template dC residue leading to pausing (Fig. 8A). The G_−10_ strongly interferes with forward translocation but allows backtracking followed by rapid cleavage of the extruded 3′ RNA in the presence of Gre factors (Fig. 8B). The rapid cleavage and the slow translocation prevent the escape of RNAP from the pre-translocated state even in the presence of Gre factors. The G_−11_ at the upstream end of the RNA-DNA hybrid is important to stabilize backtracked states. Note that G_−11_ in a 1-bp backtracked complex corresponds to G_−10_ in the pre-translocated complex (Fig. 5B). As G_−10_ inhibits forward translocation, G_−11_ inhibits conversion of the 1-bp backtracked state to the pre-translocated state, thereby favoring continued backtracking in the upstream direction. In WT cells, Gre proteins rapidly cleave the RNA in the backtracked state, allowing RNAP to enter back into the pre-translocated state with G_−10_ present. In contrast, lack of the RNA cleavage in *ΔgreAB* cells leads to a predominantly backtracked EC pool with G_−11_ present. Taken together, a bipartite pausing sequence, G_−9_, G_−10_, or G_−11_ with TGC_−1_G_+1_ can confer pauses in all translocated states of RNAP *in vivo*.

In *ΔgreAB* cells, the weak U-dA base pairs in the −4/−7 positions of the hybrid contribute to further backtracking by thermodynamically destabilizing the non-backtracked complexes [78] or via a specific conformation of the hybrid with the −4/−7 U-dA base pairs that kinetically favors backtracking (Fig. 3A). Indeed, the T_−4_ T_−7_ but not G_−11_…C_−1_G_+1_ element of the PIE was reduced in the weak backtrack pausing signals (compare the *ΔgreAB* PIEs between the high and low *ϕ* parameter; Fig. S6B in Additional file 1). The observed preference for the U-dA base pairs in the −4/−7 positions of the hybrid seems to disagree with the thermodynamic model for pausing, which suggests that the unstable U-rich RNA-DNA hybrid is merely required for RNAP backtracking [78]. Several well-characterized backtracking signals for bacterial RNAP and eukaryotic RNAPII contain the uniform runs of U residues in the RNA [20, 79, 80]. This discrepancy may indicate that *E. coli* cells have evolved not to contain strong PIEs made of a combination of T tracts followed by the C_−1_G_+1_ sequence to avoid collisions of stably backtracked RNAPs with replication machineries.

While this work was in progress, Larson *et al.* [55] reported NET-seq analysis of a consensus pausing sequence in *E. coli* that turned out to be very similar to the PIE identified by us. This group showed an enrichment of RNAP pausing at the translation start sites (i.e., the AT_−1_G_+1_ motif) in *E. coli* genes, which was interpreted as a key mechanism for synchronization of transcription and translation [55]. In contrast to their conclusion, we found only a few AT_−1_G_+1_ sequences compared with major TGC_−1_G_+1_ sequences in pause sites: seven of all the robust 758 pause sites (<1 %) that we identified by RNET-seq contained an AT_−1_G_+1_ motif and only two out of these seven AT_−1_G_+1_ pause sites were located at the ATG start codon (*lpxD* and *pgk* genes). Larson *et al.* also showed no contribution of Gre factors to pausing *in vivo* and argued that the pausing in the pre-translocated state does not accompany backtracking of RNAP [55]. In contrast, our analysis of the link between pausing and the translocation state of RNAP strongly suggests that pre-translocated pausing is typically in a dynamic equilibrium with backtracking and 3′ RNA cleavage by Gre factors (Figs. 5 and 8B). The transient pre-translocated pause equilibrated with backtracking guarantees that pausing may affect gene expression even at normal intracellular concentrations of PPi, in which the pre-translocated paused state should have a very short half-life. Indeed, our *in vitro* measurements revealed that ~100 μM PPi completes pyrophosphorolysis of the 3′ RNA base within seconds of forming the pre-translocated complex that disfavors backtracking (data not shown). Thus, any static pre-translocated pause will be rapidly reversed by pyrophosphorolysis *in vivo*, thereby grossly limiting its dynamic range and regulatory impact. Taken together, a dynamic rather than a static model for pre-translocated pauses is necessary to understand the physiologically relevant pausing.

More recently, Vvedenskaya *et al.* [81] identified a robust G_−10_Y_−1_G_+1_ pausing signal in *E. coli* by conventional NET-seq. This work employed a mutant *E. coli* strain carrying D446A substitution in the RNAP β subunit [81]. They argued that the D446 residue interacts with G_+1_ of the non-template DNA, facilitating forward translocation, which promoted read-through of paused RNAPs. However, we noted in their published data that the βD446A mutation led to a major increase in both C_−1_ and G_+1_ of the pausing motif with only a minor effect on their ratio [81]. Thus, the βD446A RNAP was sensitive to the C_−1_G_+1_ neighbor rather than to G_+1_ alone, consistent with previous extensive *in vitro* analyses of pausing signals for *E. coli* RNAP [1, 12, 66, 67]. We note that our RNET-seq analysis, in which we employed only single read lengths, detected some fluctuation in the ratio of G_+1_ to C_−1_ at pause sites depending on the RNA length in the complex. This fluctuation correlated with translocation register and the presence of Gre factors, indicating its relevance to transcription pausing (Figs. S9 and S10 in Additional file 1). This phenomenon indicates the presence of several additional pausing mechanisms related to the C_−1_G_+1_ neighbor, including the proposed interaction of the G_+1_ base in the non-template DNA strand with the D446 residue of the RNAP β subunit [81]. More analysis will be needed to elucidate the contributions of C_−1_ and G_+1_ to different classes of pausing signals.

## Conclusions

We present several pausing mechanisms governed by a bipartite RNA-DNA hybrid consensus sequence, which consists of an upstream part, a G at either −9, −10 or −11 nt from the 3′ RNA end, and a downstream part, a C_−1_G_+1_ flanking the active center. The upstream G position determines whether pausing occurs in the post-, pre-translocated or backtracked state, respectively. We suggest that pauses have multiple regulatory roles during transcription of the 5′ UTR regions of genes and during antisense transcription. Using this mechanism, RNAP pausing and its regulation by *trans*-acting factors can be optimized to suit different genomes with different GC content and CpG repeats that are broadly present in eukaryotes [82]. Backtracking of RNAP at these sequences imposes a strong roadblock to DNA replication leading to DNA double strand breaks [21]. By rescuing the backtrack pauses, Gre/TFIIS factors would be essential for maintaining genome integrity as suggested previously [1, 25, 83, 84].

We also reveal that DNA sequences for the predominant G-to-A transcription errors in *E. coli* coincide with the CpG pausing motif throughout the *E. coli* genome. Thus, the CpG-enriched domain found in chromosomes may play a special role associated with prolonged RNAP backtracking in combination with an elevated error frequency. The CpG domain may compromise genome integrity due to elevated rates of collisions between replication and backtracked transcription complexes via stable R-loop formations [22, 24]. It has been shown that R-loops are increased in unmethylated human CpG islands [85]. Pausing sequences identified for human RNAPII *in vitro* are similar to the PIE in *E. coli* [66]. Thus, transcription pausing followed by the G-to-A error may be a source of the R-loops in CpG islands and transcription of these islands may significantly contribute to transcription mutagenesis in both prokaryotes and eukaryotes [86].

## Materials and methods

### Bacterial culture

*E. coli* cells carrying the *rpoC* gene coding for the C-terminal-hexahistidine-tagged β’ subunit, NB854 (W3110 *rpoC*-6xHis::*kan gal490*) or NB959 (W3110 *rpoC*-6xHis::*kan greA*::*tet*, *greB*::*amp*) were constructed by λRed-mediated recombination (see Method S2 in Additional file 3 for detail). To prepare WT and *ΔgreA/greB* cells for extraction and isolation of RNAP complexes, cells were grown in ~300 ml LB broth +25 μg/ml kanamycin with shaking at 37 °C until an OD_600_ of ~0.5. The cells (150 mg wet weight) were harvested by centrifugation at 6,500 × *g* for 4 minutes at 4 °C, divided into three 1.5 ml tubes, flash frozen in liquid nitrogen, and stored at −80 °C.

### Rapid breakdown of cells and nucleoids

Each tube of the cell pellet stored at −80 °C was resuspended in 650 μl TES buffer (10 mM Tris–HCl, pH 7.5, 1 mM EDTA, 100 mM NaCl, 0.1 % TritonX 100 and 0.2 mM PMSF) at room temperature. The cell suspension was mixed with 100 kU Ready-Lyse Lysozyme (Epicentre) and 50 μg RNase A (Sigma) and incubated for 5 minutes, allowing rapid cell lysis. To digest the genomic DNA, 62.5 U of DNase I (Roche) was added with heparin at a final concentration of 250 μg/ml and 74 μl of 10 x DNase I buffer (100 mM MnCl_2_ and 100 mM Tris–HCl, pH 7.5). All were added to the mixture and incubated for 10 minutes at room temperature. To remove cell debris, the mixture was centrifuged at 20,000 × *g* for 3 minutes at 4 °C and the supernatant was collected into a new 1.5 ml tube.

### Purification of elongation complexes

The supernatant including ECs were immobilized on a 50 % suspension of ~155 μl Ni^2+^-NTA agarose (Qiagen) pre-equilibrated with the binding buffer (0.5 M NaCl, 5 mM imidazole, 5 % glycerol, 20 mM Tris–HCl, pH 7.9 and 1 mM 2-mercaptoethanol) with shaking for 10 minutes at 4 °C. The immobilized ECs were washed at 4 °C five times with the wash buffer (1 M NaCl, 15 mM imidazole, 5 % glycerol, 20 mM Tris–HCl, pH 7.9 and 1 mM 2-mercaptoethanol), and then washed twice with the nuclease buffer (40 mM KCl, 15 mM imidazole, 20 mM Tris–HCl, pH 7.9, 0.3 mM MgCl_2_, 5 % glycerol and 1 mM 2-mercaptoethanol). Any DNA unprotected by RNAP was cleaved by the addition of 0.4 U RNase V1 (Invitrogen), 0.7 U RNase T1 (Sigma), and 5 U DNase I (Takara Bio) to the immobilized complexes with incubation for 7 minutes at room temperature. The nuclease-treated complexes were washed twice with the wash buffer, twice with the MgCl_2_-free nuclease buffer at 4 °C, and eluted by adjusting the concentration of imidazole to 100 mM in the presence of 30 U SUPERase RNase inhibitor (Ambion) followed by shaking for 10 minutes at 4 °C.

To test the functional RNAP activity of the purified ECs, 5 mM MgCl_2_ and 0.5 mM NTP ± 8 μM GreA and 4 μM GreB were added to the complexes (not used for RNET-seq) and incubated for 5 minutes at 37 °C to follow RNAP transcription. To visualize RNA and DNA species that were associated with the ECs, the samples were heat-denatured for 10 minutes at 70 °C and either 50 U/ml DNase I or 5 μg/ml RNase A was added to the sample to digest the DNA or RNA in the sample, respectively. The samples were subjected to PAGE with 15 % TBE-Urea gels (Invitrogen) followed by staining with SYBR Gold (Invitrogen) according to [87] (Fig. S1B in Additional file 1). ECs carrying ~14- to 20-nt long transcripts resumed elongation when incubated *in vitro* with NTPs and purified GreA and GreB proteins (Fig. S1B in Additional file 1). The fraction of ECs that resumed transcription after treatment with Gre protein appeared to be larger in *ΔgreAB* compared with WT cells (Fig. S1C in Additional file 1). A substantial fraction of the initiation complexes carrying RNAs ≤13 nt required Gre proteins to resume transcription in both WT and *ΔgreAB* cells. This fraction may include moribund initiation complexes that require Gre proteins to enter productive elongation [39, 48].

### RNA extraction

The 200 μl eluate from the Ni^2+^-NTA agarose was mixed with an equal volume of pre-warmed phenol/chloroform/isoamylalcohol (PCI; 25:24:1) and incubated for 2 minutes at 70 °C. The mixture was centrifuged, and RNA and DNA were precipitated with isopropanol from the supernatant according to [87]. The pellet was dissolved in 30 μl DNase I buffer with 5 U DNase I (Takara Bio) and 20 U SUPERase and incubated for 10 minutes at room temperature. RNA was separated from the digested DNA by the PCI extraction and the RNA was precipitated by isopropanol. The pellet was dissolved in diethylpyrocarbonate-treated water and used for cDNA synthesis.

### Library construction and sequencing

cDNA libraries of the nascent RNAs were constructed according to [87]. Briefly, a pre-adenylated DNA linker was ligated onto ~1 μg of the purified nascent RNA. The RNA-fragmentation step was skipped in our protocol. Reverse transcription was performed with a DNA primer containing the linkers specific for Illumina sequencing and carbon spacers (Integrated DNA Technologies) and an enzyme PrimeScript (Takara Bio). The resulting single-stranded DNA was circularized with DNA CircLigase (Epicentre), and used as a template for PCR. Eleven cycles of PCR were performed with an enzyme PrimeSTAR Max (Takara Bio), which produced the double-stranded DNA ready for sequencing with the Illumina platform. Quantification of the cDNAs and Illumina sequencing were performed as described previously [25], except that a GAIIx single-end run with 36 bp length was employed.

### Data analysis

The fastq files of 36-bp sequenced reads were generated with CASAVA v1.8 (Illumina). For the bulk RNET-seq analysis, the specific adapter sequences were trimmed with Trimmomatic v.0.25 [88] to obtain reads ≥21 nt from the 5′ end. The reads ≥21 nt were mapped to the reference genome of *E. coli* K-12 strain W3110 (NC_007779.1) [or MG1665 (NC_000913.2) when transcription start site information was necessary for the sequence analysis], using Bowtie2 v.2.1.0 with default parameters [89]. We verified high Phred quality scores *Q* through the mapped reads in both WT and *ΔgreAB* strains (Fig. S3 in Additional file 1; *Q*_median_ = 38 and *Q*_lowest_ > 28) [90]. The RNA read sequences were not always uniquely mapped and could be found at multiple locations on the *E. coli* genome. We arbitrarily included reads that were mapped to multiple chromosomal locations (~40 % of the total mapped reads for the bulk RNET-seq analysis) because both their inclusion and exclusion would generate artifacts. We showed that such multiple mapping events were highly enriched in rRNA genes and IS elements (Fig. S7 in Additional file 1). To analyze RNAP pausing on the *E. coli* chromosome, we counted the number of reads at every genomic nucleotide position using the mpileup command of SAM tools v.0.1.18 with –A –B parameters [91]. Pausing sites were defined as described in the main text and the legend of Fig. 3A. The information contents of PIEs were visualized using WebLogo [51, 92]. The bulk RNET-seq also found a single rRNA sequence, which was the same for both WT and *ΔgreAB* cells. Its sequence was completely different from either PIE determined for mRNA genes (Fig. 3A; Fig. S7 in Additional file 1). The sequencing reads for this RNA also had an extraordinarily high (~3 × 10^6^) read depth, suggesting presence of a very strong pause at this location or, more likely, formation of a stable binary complex of RNAP with this rRNA fragment *in vivo* or during the RNase treatment.

For single-length RNET-seq analyses, all sequence reads were sorted by their length into 6 nt to 30 nt. The reads were mapped to the reference sequence (NC_007779.1) as described above. The distribution of each read length is shown in Fig. 1B. In the PIEs for ~14- to 19-nt reads, single-length RNET-seq analysis found an enrichment of G residues at the 3′ cleavage site generated by RNases used for the 5′ RNA trimming (Figs. S9 and S10 in Additional file 1). Because RNaseT1 specifically cuts GpN bonds in the RNA, whereas RNaseA cuts CpN or UpN bonds, a large fraction of RNA species of ~14–19 nt had been cleaved at GpN, the signature of an RNaseT1 cut (Figs. S9 and S10 in Additional file 1). This indicates that after the RNase treatment for ECs, the canonical assignment where 14-nt RNA corresponds to the post-translocated state, was made mostly when a G residue was positioned immediately 5′ of the nascent RNA protected by RNAP. Consequently, this bias underestimated the presence *in vivo* of the post-translocated state (14 nt) primarily and that of the pre-translocated state (15 nt) secondly as opposed to the backtracked states (>15 nt). For this reason, 14- to 18-nt reads were used for obtaining information about translocated/backtracked states in paused RNAP *in vivo*, and ≥21-nt reads lacking an obvious signature of the RNaseT1 cut (no signature of translocated/backtracked states) were used for a genome-wide analysis of pausing pattern.

The sequence reads for each gene were determined along the length of the gene using the HTSeq v.0.5.4p2 [93]. A gene annotation file of *E. coli* W3110 was downloaded from the ftp server of Ensembl [94]. To accommodate the seven nearly identical rRNA genes, we divided the read depths found in rRNA genes by seven to represent the average of one rRNA operon. Normalization of read counts for each gene or each gene body in the bulk RNET-seq analysis was performed by 10^6^ × (Sum of reads)/Total mapped reads (equal to 6,967,786 for WT or 11,174,399 for *ΔgreAB*). The transcription start site and operon data sets in *E. coli* were downloaded from RegulonDB [95]. For the transcription start site, we used positions having the maximum transcription start site frequency in the high-throughput transcription initiation mapping (version 3.0). The error rate per read position was calculated by counting each type of error in each read position using CIGAR and MD:Z tags of SAM format with a Perl script. All the Perl scripts developed and used in this study are publicly available at [96].

### Data availability

Raw sequencing data and processed data are available at the Gene Expression Omnibus under accession number GSE62102 [97].

## Additional files

Additional file 1: Figure S1.
**A** Purified RNA used for the RNET-seq analysis in *E. coli* WT and *ΔgreAB* cells. **B** Elongation activities of the purified initiation/elongation complexes with and without Gre proteins (see “Materials and methods” for details). **C** Quantification of the gel images shown in Fig. S1B. **Figure S2.** Short reads derive from initiation complexes. The information about the transcription start sites was obtained as described in Materials and methods. **Figure S3.** Phred quality scores per position through 21-nt reads. **Figure S4. A** Genome-wide gene-specific comparisons of the bulk RNET-seq results between *E. coli* WT and *ΔgreAB* cells. **B** Gre-dependent suppression of pausing in the Up region is correlated with 5′ UTR lengths close to 50 bases. **Figure S5.** Cartoon images for pausing and non pausing when defined by *P*(0.9, 100) or *P*(0.9, 160). The latter is shown in parentheses. **Figure S6. A** The PIEs for WT and ΔgreAB strains (Fig. 3A) are unique. **B** The PIEs identified by bulk RNET-seq are robust against parameters *ϕ* and *δ*. **Figure S7.** Mapping quality (mapq) controls of the pause sites in WT (*top*) and *ΔgreAB* (*bottom*) cells. The mapq is defined as −10 log_10_p, where p is an estimate of the probability that the read mapped to a position(s) in addition to the particular position [57]. **Figure S8. A** Scatter plot of antisense and sense transcription in WT strain. **B** Antisense transcriptional pauses observed in the *insAB* genes of WT cells. **Figure S9.** PIEs determined by single-length RNET-seq in WT strain. **Figure S10.** PIEs determined by single-length RNET-seq in *ΔgreAB* strain. **Figure S11. A** Box plots of mapq_mean_ for the pause sites identified by single-length RNET-seq analysis using 14-, 15- or 16-nt reads. **B**G_−9_ is favored for the pause sites with low mapping quality in 14-nt reads. **Fig. S12** Effect of 5-methyl-dC_+1_ in the template DNA on RNAP translocation *in vitro*. **Figure S13.** Error rates per position in nascent transcripts detected by single-length (14–18 nt) RNET-seq. Additional file 2: Table S1. Frequency matrix, position weight matrix and MAP scores for PIEs. **Table S2.** MAP scores for transcription factors in *E. coli*. Additional file 3:
** Method S1.** Calculation of MAP scores for PIEs. **Method S2.** Inactivation of *greA* and *greB* genes.

## Abbreviations

bp: base pair
CDS: coding DNA sequence
EC: elongation complex
IS: insertion sequence
NET-seq: native elongating transcript sequencing
nt: nucleotide
NTP: ribonucleoside triphosphate
PIE: pause-inducing element
PPi: pyrophosphate
RNAP: RNA polymerase
RNET-seq: RNase-footprinting followed by NET-seq
UTR: untranslated region
WT: wild type
