# Erratum to: Visualizing translocation dynamics and nascent transcript errors in paused RNA polymerases *in vivo*

**DOI:** 10.1186/s13059-015-0845-4

**Published:** 2015-12-02

**Authors:** Masahiko Imashimizu, Hiroki Takahashi, Taku Oshima, Carl McIntosh, Mikhail Bubunenko, Donald L. Court, Mikhail Kashlev

**Affiliations:** Center for Cancer Research, National Cancer Institute, Frederick, 21702 MD USA; Medical Mycology Research Center, Chiba University, 1-8-1 Inohana, Chuo-ku, 260-8673 Chiba Japan; Graduate School of Biological Sciences, Nara Institute of Science and Technology, 8916-5, Ikoma, 630-0192 Nara Japan

After the publication of this work [1], we noticed an error whereby Fig. 3b is missing the label ΔgreAB. The original version of this article was corrected. The corrected figure is given below:

**Fig. 3:**
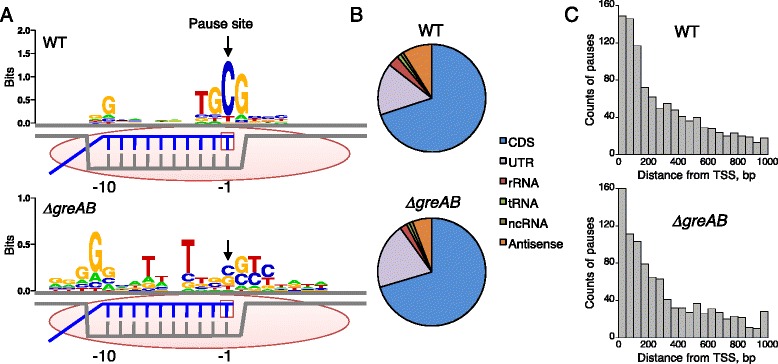
Transcription pausing detected by RNET-seq in *E. coli* WT and Δ*greAB* cells. (**a**) Pause-inducing elements (PIEs) of the non-template DNA strand. Information content is represented by sequence logos [51]. Positions −1 and −10 of DNA (*gray*) correspond to the RNA (*blue*) 3′ and 5′ ends of the RNA-DNA hybrid within RNAP (*pink oval*) in pre-translocated ECs. The active site is shown as an *empty square. P*(0.9, 100) and *P*(0.9, 160) were used for WT (*n* = 758) and Δ*greAB* (*n* =419), respectively (see main text for the parameters). A frequency matrix and MAP scores for the PIEs are shown in Table S1 in Additional file 2. (**b**) Categorization of all RNAP pauses by RNA type. The non-coding RNA (*ncRNA*) and antisense RNA were defined using the gene annotation file of *E. coli* (see “Materials and methods”). (**c**) Pausing frequently occurs in regions proximal to transcript start sites (*TSS*). For panels B and C, *P*(0.7, 100) is used in order to increase the number of samples. Note that even when using this reduced stringency the consensus sequence for pausing remains unaffected (Fig. S6B in Additional file 1)

## References

[1] Imashimizu M, Takahashi H, Oshima T, McIntosh C, Bubunenko M, Court DL (2015). Visualizing translocation dynamics and nascent transcript errors in paused RNA polymerases in vivo. Genome Biol..

