# Supplementary material for: Visualizing translocation dynamics and nascent transcript errors in paused RNA polymerases in vivo
Source: Genome Biol. 2015 May 15;16(1):98. doi: 10.1186/s13059-015-0666-5 (PMC4457086; doi:10.1186/s13059-015-0666-5)
Supplement: Additional file 1: Figure S1. — A Purified RNA used for the RNET-seq analysis in E. coli WT and ΔgreAB cells. B Elongation activities of the purified initiation/elongation complexes with and without Gre proteins (see “Materials and methods” for details). C Quantification of the gel images shown in Fig. S1B. Figure S2. Short reads derive from initiation complexes. The information about the transcription start sites was obtained as described in Materials and methods. Figure S3. Phred quality scores per position through 21-nt reads. Figure S4. A Genome-wide gene-specific comparisons of the bulk RNET-seq results between E. coli WT and ΔgreAB cells. B Gre-dependent suppression of pausing in the Up region is correlated with 5′ UTR lengths close to 50 bases. Figure S5. Cartoon images for pausing and non pausing when defined by P(0.9, 100) or P(0.9, 160). The latter is shown in parentheses. Figure S6. A The PIEs for WT and ΔgreAB strains (Fig. 3A) are unique. B The PIEs identified by bulk RNET-seq are robust against parameters ϕ and δ. Figure S7. Mapping quality (mapq) controls of the pause sites in WT (top) and ΔgreAB (bottom) cells. The mapq is defined as −10 log10p, where p is an estimate of the probability that the read mapped to a position(s) in addition to the particular position [57]. Figure S8. A Scatter plot of antisense and sense transcription in WT strain. B Antisense transcriptional pauses observed in the insAB genes of WT cells. Figure S9. PIEs determined by single-length RNET-seq in WT strain. Figure S10. PIEs determined by single-length RNET-seq in ΔgreAB strain. Figure S11. A Box plots of mapqmean for the pause sites identified by single-length RNET-seq analysis using 14-, 15- or 16-nt reads. BG−9 is favored for the pause sites with low mapping quality in 14-nt reads. Fig. S12 Effect of 5-methyl-dC+1 in the template DNA on RNAP translocation in vitro. Figure S13. Error rates per position in nascent transcripts detected by single-length (14–18 nt) RNET-seq. [file 13059_2015_666_MOESM1_ESM.pdf]

Figure S1

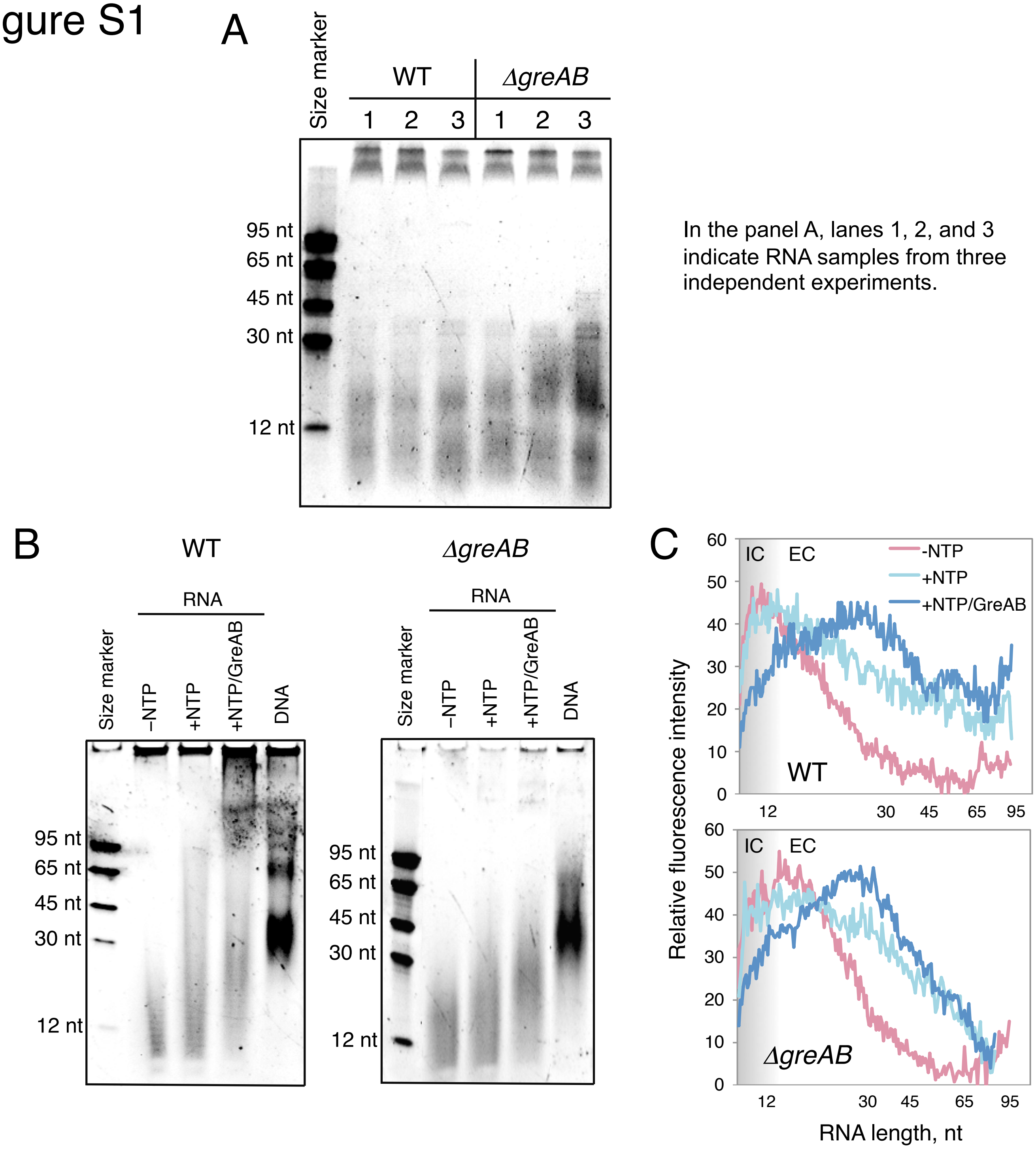

In the panel A, lanes 1, 2, and 3 indicate RNA samples from three independent experiments.

In the panel B, some fraction of backtracked ECs that required GreAB for elongation may be caused by the RNase digestion of the secondary structure in the 5′ nascent RNA. These structures have been shown to interfere with RNAP backtracking [1]. The digestion of the complexes with DNase I produced a major ~35-bp and a minor ~65-bp peak, which likely represented the DNA regions protected by one or two RNAP molecule(s), respectively. [1]. In the panel C, the purified initiation complexes (IC) are shaded.

1. Komissarova N, Kashlev M. Transcriptional arrest: Escherichia coli RNA polymerase translocates backward, leaving the 3′ end of the RNA intact and extruded. Proc Natl Acad Sci. 1997;94:1755–60.

Figure S2

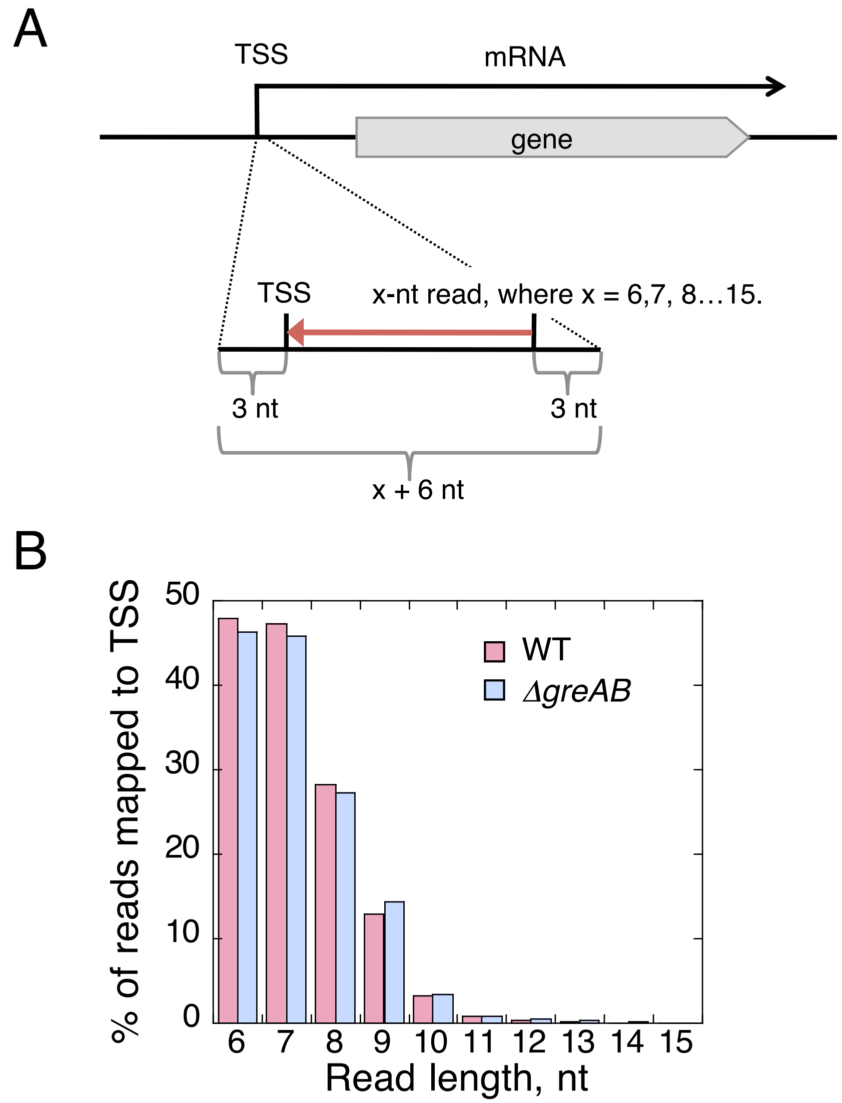

(**A**) For the short reads mapping, we made the special reference sequences containing only 3nt upstream and N + 3nt downstream from the transcription start site, where N is the read length in a range of 6-15 nt. The short reads of each length with sense orientation to mRNA genes were mapped to the references for perfect matches by Blat (Standalone BLAT v. 35x1). (**B**) Counts for the mapped reads are represented as histograms.

Figure S3

WT

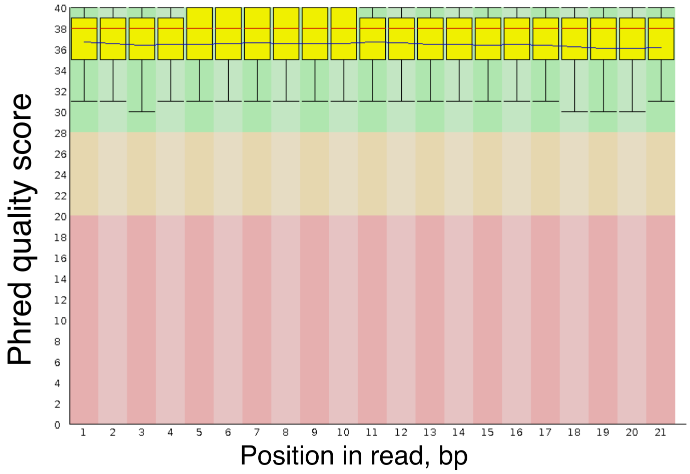

ΔgreAB

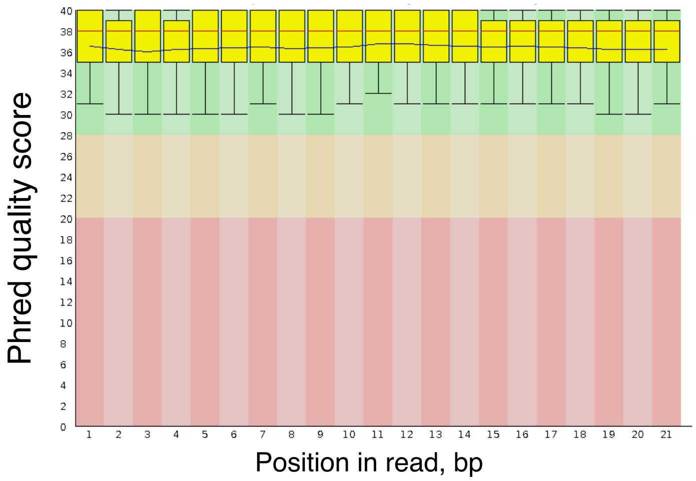

Box plots were generated using the program FastQC (http://www.bioinformatics.babraham.ac.uk/projects/fastqc/).

Figure S4

A

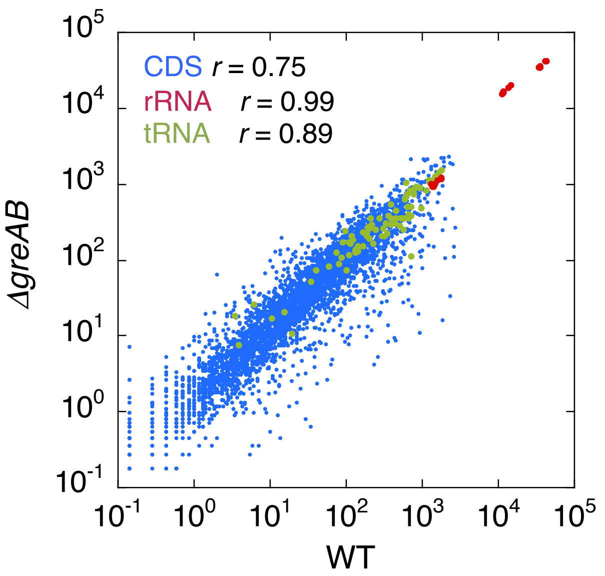

Scatter plot of the normalized read counts in each gene with Pearson's correlation coefficient (*r*) are shown.

B

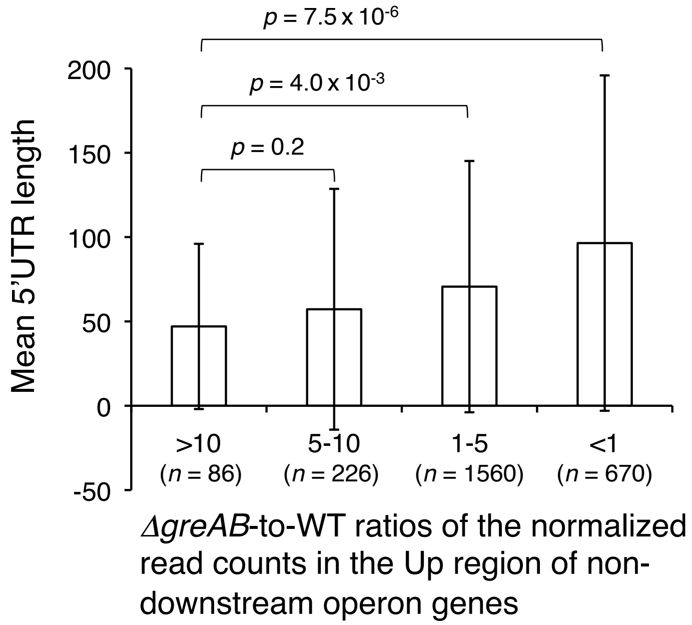

The 5′UTR lengths were obtained from the reference [49]. The mean values for 5′UTR lengths ± standard deviations, the number of samples (*n*) and the *p*-values of two-tailed t-test for pairs are shown.

Figure S5

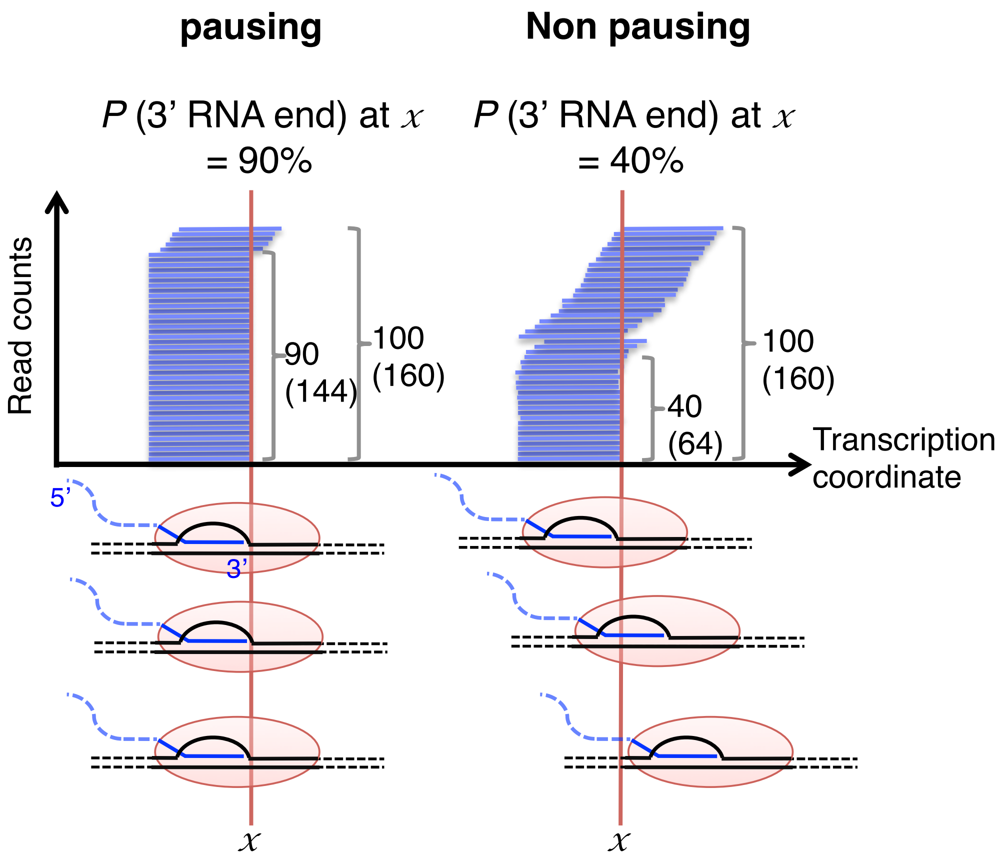

If RNAPs pause at a position X (left), the 3′ ends of RNAs associated with these RNAPs (oval shapes) are aligned at the position X. If RNAPs do not pause (right), the 3′ ends spread across the position X.

Figure S6

A

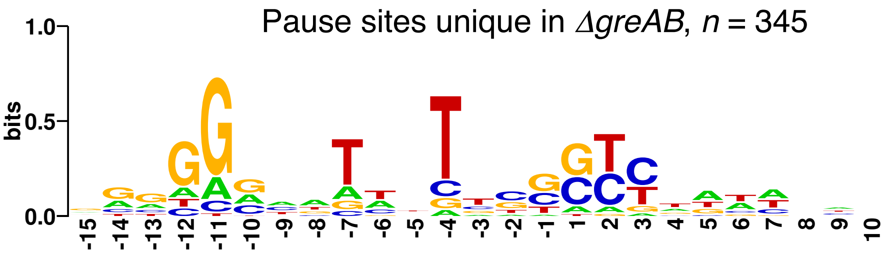

The PIE unique for *ΔgreAB* pause sites was obtained by subtracting identical pause sites between WT and *ΔgreAB* datasets.

B

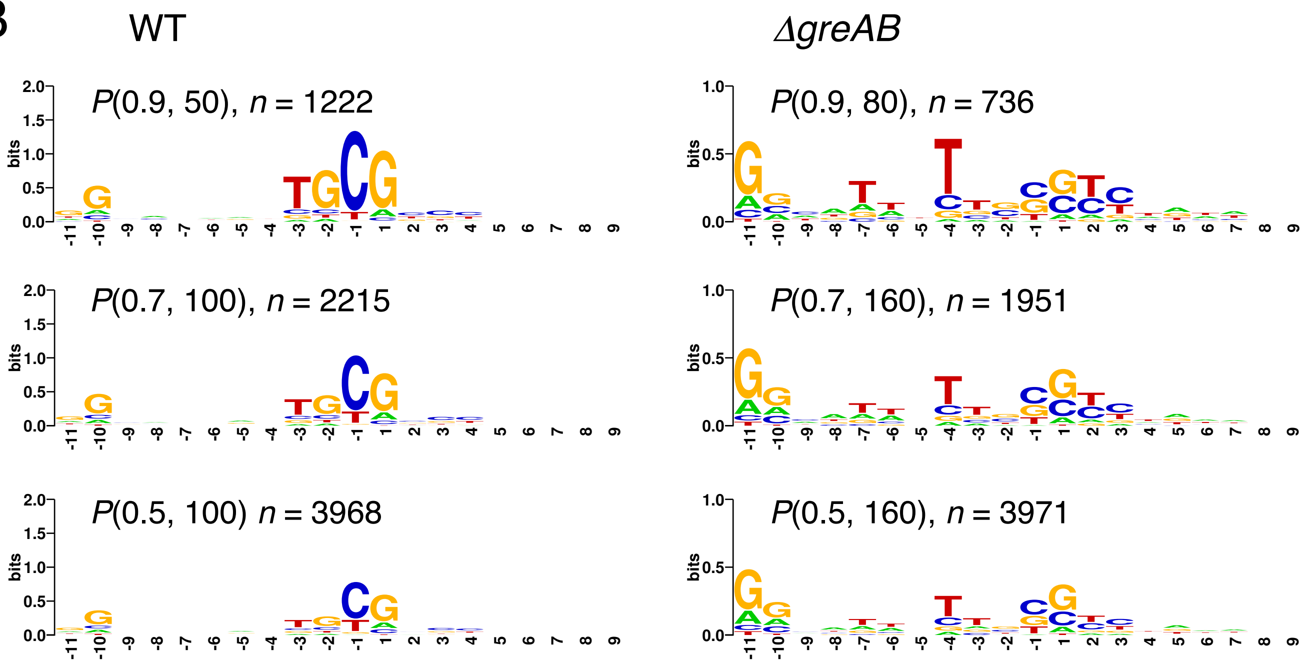

Figure S7

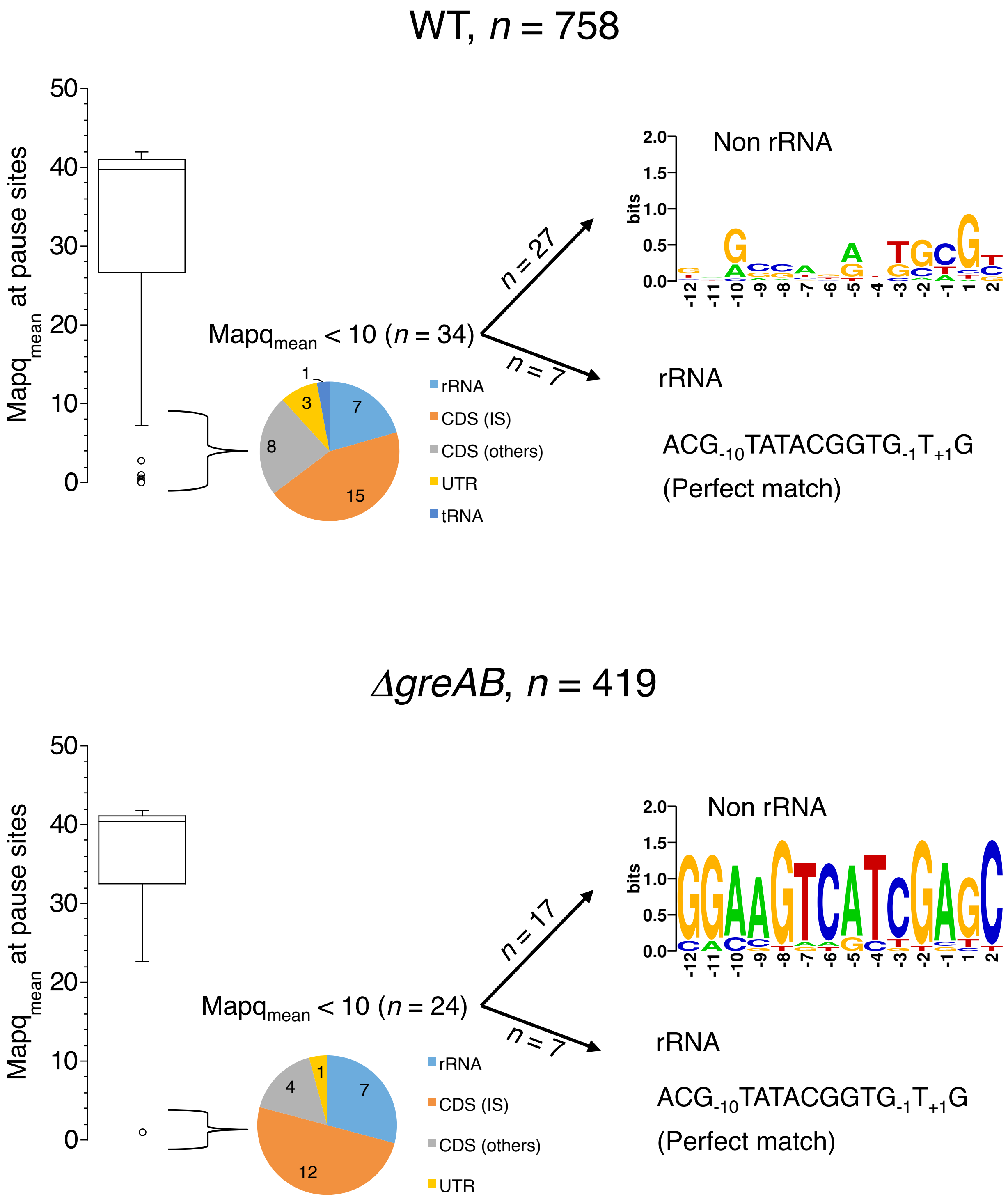

When the $mapq_{mean}$ = 10 at a pause site, one of ten reads was mapped somewhere in the genome besides the particular pause site [57]. The $mapq_{mean}$ scores for all pause sites shown in Fig. 3A were calculated with the SAM formats and the quartiles are represented by box plots. The PIEs with $mapq_{mean}$ < 10 (non-rRNA or rRNA genes) are also shown.

Figure S8

A

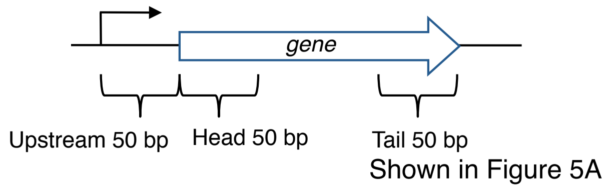

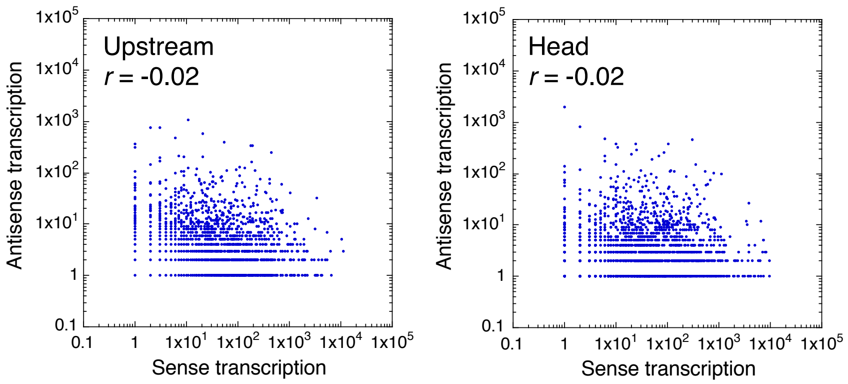

Each dot represents each gene of upstream (left) and head (right) region in 50-bp window. Pearson's correlation coefficient (r) is shown.

B

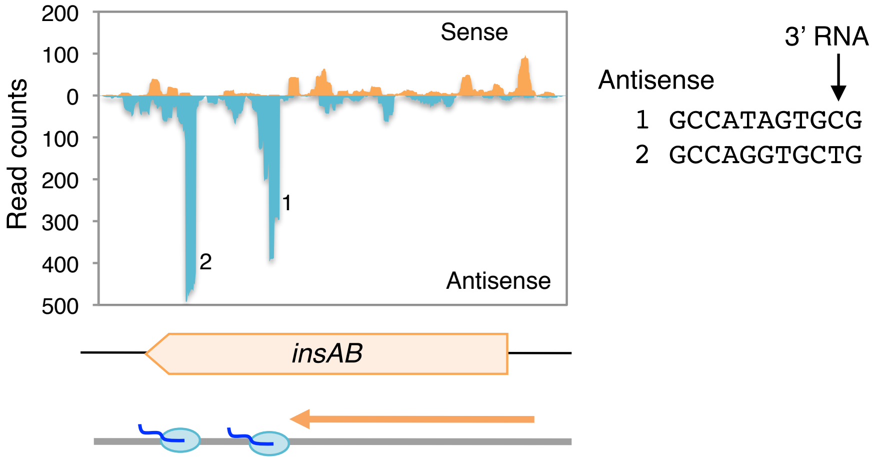

The sequences for the two strongest antisense pause sites (labeled by 1 and 2) are shown. The other symbols are the same as in Fig. 4B.

Figure S9

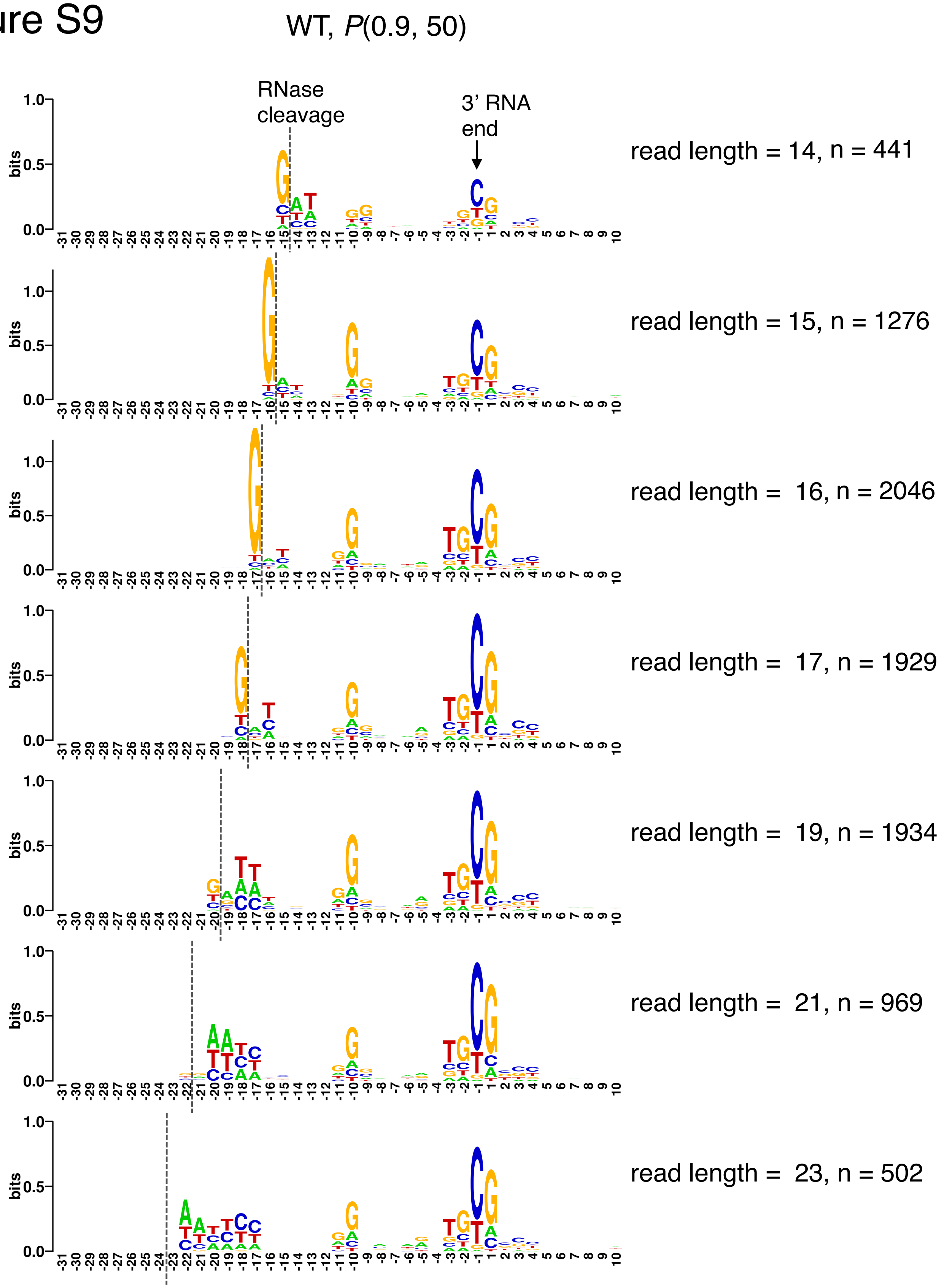

Pausing was defined by *P*(0.9, 50). The RNase-cleavage sites and the 3′ RNA sites are shown on the top. The read length and the number of samples (n) are shown on the right side.

Figure S10

ΔgreAB, *P*(0.9, 50)

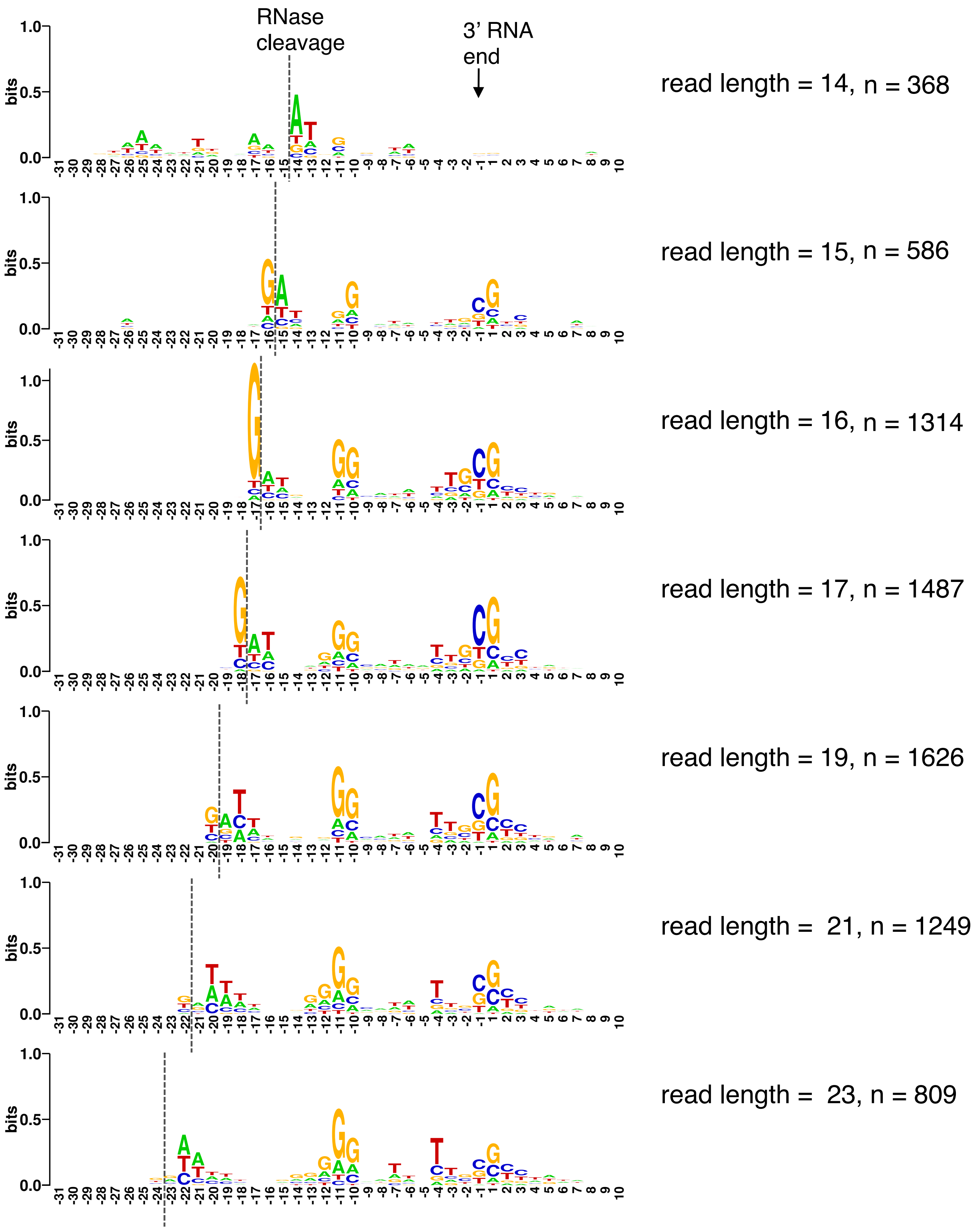

Pausing was defined by *P*(0.9, 50). The RNase-cleavage sites and the 3′ RNA sites are shown on the top. The read length and the number of samples (n) are shown on the right side.

Figure S11

A

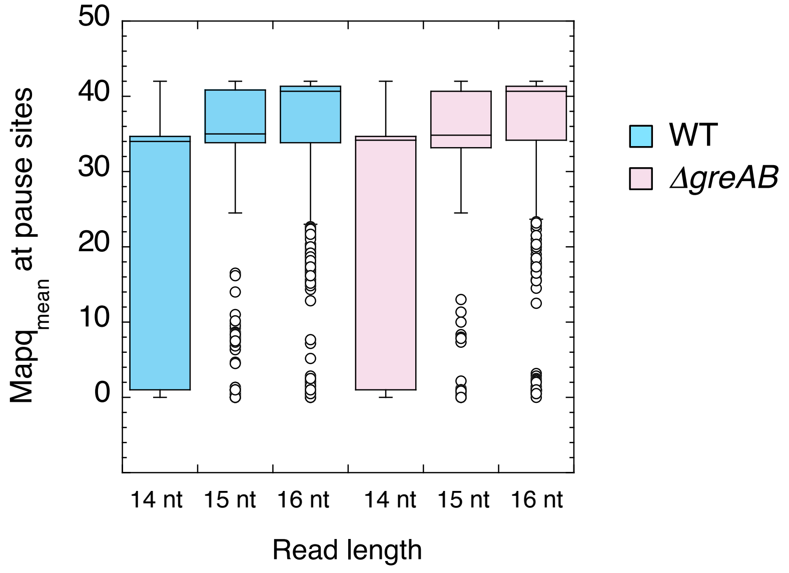

B

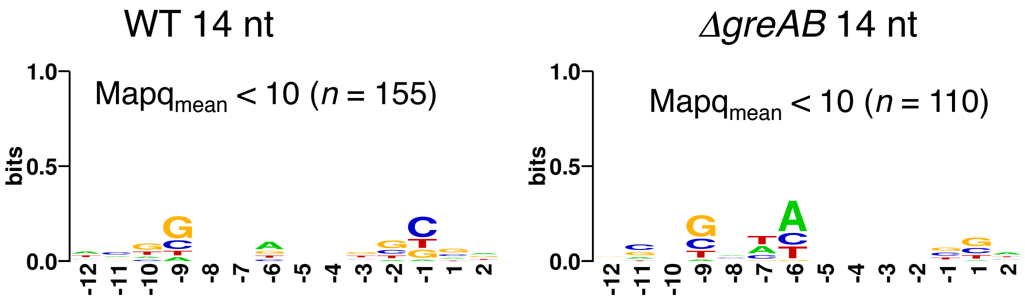

Figure S12

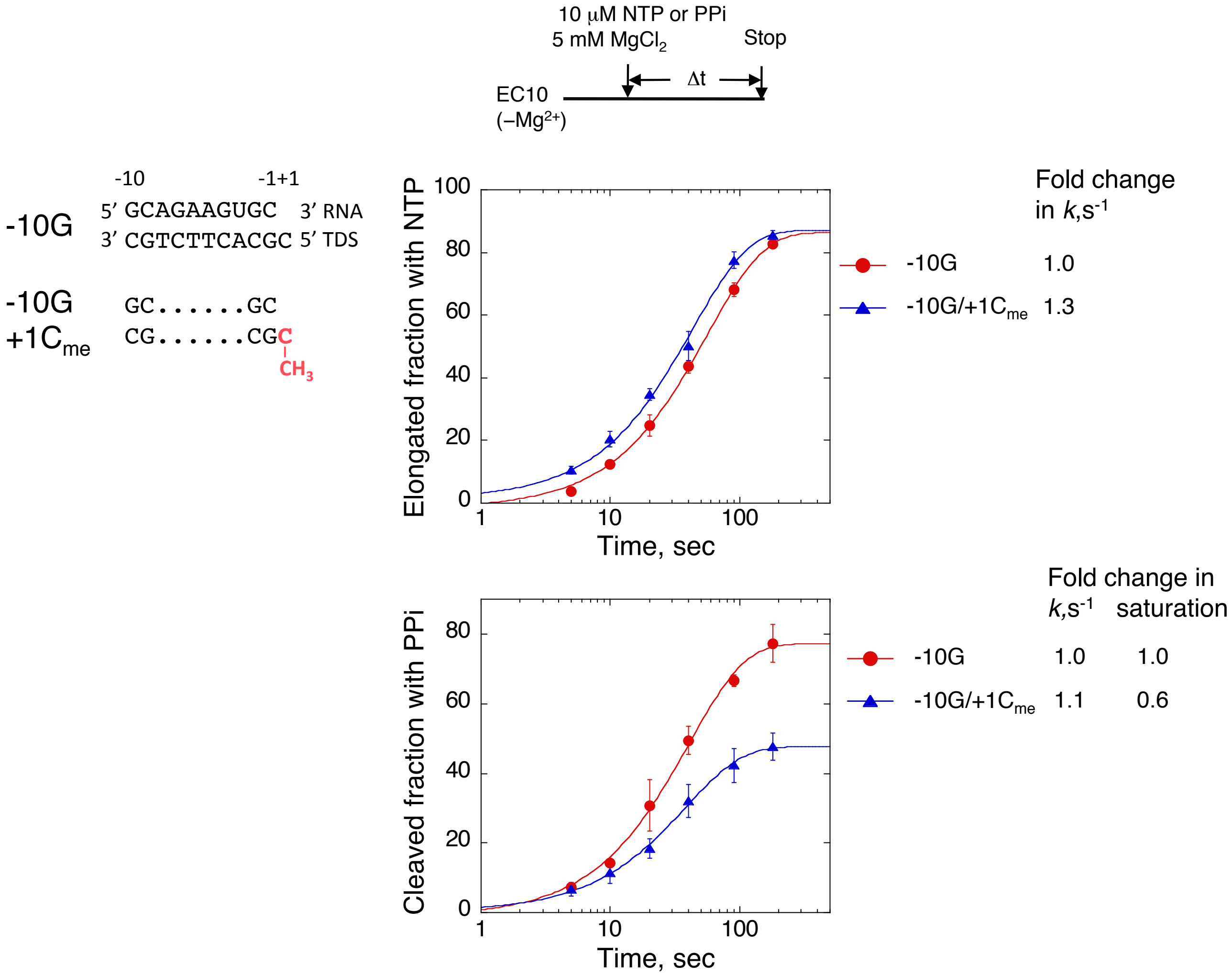

Legends are the same as in Figure 5C and D.

Figure S13

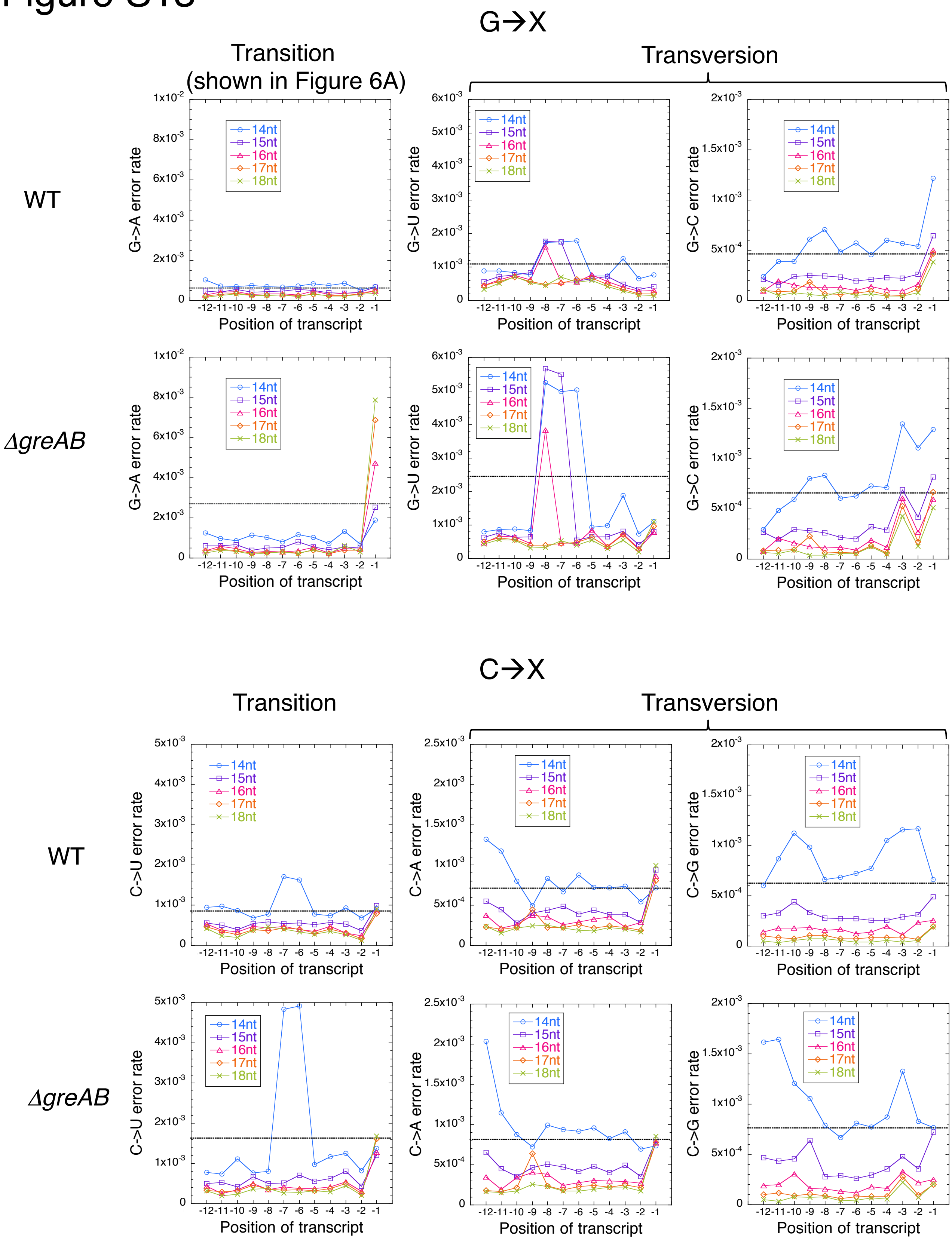

Legends are the same as in Figure 6A.

Figure S13 (continued)

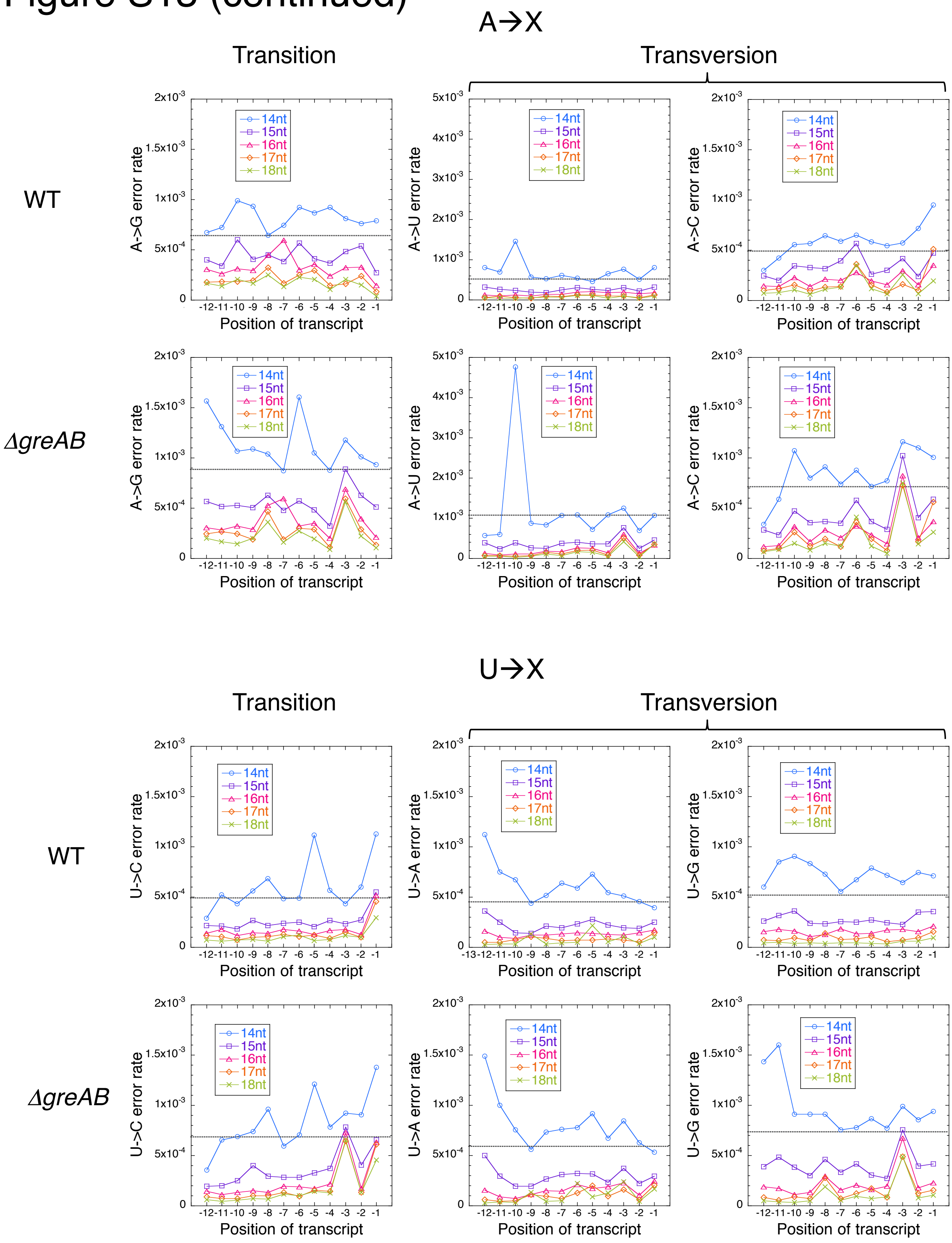

Legends are the same as in Figure 6A.
