# Supplementary material for: Visualizing translocation dynamics and nascent transcript errors in paused RNA polymerases in vivo
Source: Genome Biol. 2015 May 15;16(1):98. doi: 10.1186/s13059-015-0666-5 (PMC4457086; doi:10.1186/s13059-015-0666-5)
Supplement: Additional file 2: Table S1. — Frequency matrix, position weight matrix and MAP scores for PIEs. Table S2. MAP scores for transcription factors in E. coli. [file 13059_2015_666_MOESM2_ESM.pdf]

Table S1. Frequency matrix, position weight matrix and the MAP score for the PIE.

WT

GC contents

A	0.246
C	0.254
G	0.254
T	0.246

Frequency Matrix
758 sequences

**Position**	**-10**	**-9**	**-8**	**-7**	**-6**	**-5**	**-4**	**-3**	**-2**	**-1**	**1**
**A**	101	141	286	169	261	266	112	42	49	8	78
**C**	102	272	229	150	109	136	221	90	69	673	42
**G**	511	229	143	183	143	243	184	80	572	14	624
**T**	44	116	100	256	245	113	241	546	68	63	14

Position Weight Matrix

**Position**	**-10**	**-9**	**-8**	**-7**	**-6**	**-5**	**-4**	**-3**	**-2**	**-1**	**1**
**A**	0.133	0.186	0.377	0.223	0.344	0.351	0.148	0.056	0.065	0.011	0.103
**C**	0.135	0.359	0.302	0.198	0.144	0.180	0.292	0.119	0.091	0.887	0.056
**G**	0.674	0.302	0.189	0.241	0.189	0.320	0.243	0.106	0.754	0.019	0.822
**T**	0.058	0.153	0.132	0.338	0.323	0.149	0.318	0.720	0.090	0.083	0.019

p*log(p/b)

	**-10**	**-9**	**-8**	**-7**	**-6**	**-5**	**-4**	**-3**	**-2**	**-1**	**1**
**A**	-0.118	-0.075	0.232	-0.032	0.167	0.180	-0.109	-0.119	-0.125	-0.049	-0.129
**C**	-0.123	0.179	0.075	-0.071	-0.118	-0.090	0.058	-0.130	-0.135	1.600	-0.122
**G**	0.948	0.075	-0.081	-0.018	-0.081	0.108	-0.016	-0.134	1.183	-0.071	1.394
**T**	-0.121	-0.105	-0.119	0.154	0.127	-0.108	0.117	1.115	-0.131	-0.130	-0.070

**MAP**	4.987
**MAP*log(758)**	47.7
**MAP*log(758)/11**	4.337

p: position frequent probability
b: background probability (GC contents)

ΔgreAB

GC contents

A	0.246
C	0.254
G	0.254
T	0.246

Frequency Matrix
419 sequences

	**-12**	**-11**	**-10**	**-9**	**-8**	**-7**	**-6**	**-5**	**-4**	**-3**	**-2**	**-1**	**1**	**2**	**3**
**A**	72	66	103	128	176	76	150	85	24	59	45	24	42	36	36
**C**	66	45	105	133	63	37	76	102	69	85	144	171	140	156	207
**G**	223	286	189	52	85	64	39	98	55	88	160	159	213	29	56
**T**	58	22	22	106	95	242	154	134	271	187	70	65	24	198	120

Position Weight Matrix

	**-12**	**-11**	**-10**	**-9**	**-8**	**-7**	**-6**	**-5**	**-4**	**-3**	**-2**	**-1**	**1**	**2**	**3**
**A**	0.172	0.158	0.246	0.305	0.420	0.182	0.358	0.203	0.058	0.141	0.108	0.058	0.101	0.086	0.086
**C**	0.158	0.108	0.251	0.317	0.151	0.089	0.182	0.243	0.165	0.203	0.343	0.408	0.334	0.372	0.493
**G**	0.532	0.682	0.451	0.124	0.203	0.153	0.093	0.234	0.132	0.210	0.382	0.379	0.508	0.070	0.134
**T**	0.139	0.053	0.053	0.253	0.227	0.577	0.367	0.320	0.646	0.446	0.167	0.155	0.058	0.472	0.286

p*log(p/b)

	**-12**	**-11**	**-10**	**-9**	**-8**	**-7**	**-6**	**-5**	**-4**	**-3**	**-2**	**-1**	**1**	**2**	**3**
**A**	-0.089	-0.101	0.000	0.095	0.323	-0.080	0.193	-0.056	-0.121	-0.113	-0.128	-0.121	-0.130	-0.130	-0.130
**C**	-0.108	-0.133	-0.005	0.102	-0.114	-0.135	-0.088	-0.015	-0.103	-0.066	0.150	0.278	0.132	0.205	0.473
**G**	0.566	0.971	0.373	-0.128	-0.066	-0.112	-0.135	-0.028	-0.125	-0.057	0.224	0.219	0.507	-0.130	-0.124
**T**	-0.115	-0.117	-0.117	0.010	-0.027	0.709	0.212	0.121	0.899	0.382	-0.093	-0.103	-0.121	0.444	0.063

**MAP 15bp**	4.089
**MAP*log(419)**	35.62
**MAP*log(419)/15**	2.374
**MAP 13bp**	3.553
**MAP*log(419)**	30.95
**MAP*log(419)/13**	2.381

p: position frequent probability
b: background probability (GC contents)

Table S2. The MAP scores for transcription factors in *E. coli*.

Transcription Factor	MAP*log(n)/m
NsrR	5.24
NarL	5.04
FNR	4.93
PurR	4.92
Fur	4.66
LexA	4.54
PIE (11 nt) in WT strain	4.34
MetJ	4.19
IHF	4.19
CRP	4.02
Cra	3.96
MalT	3.84
ArcA	3.83
DnaA	3.77
NarP	3.69
ArgR	3.68
CpxR	3.52
Fis	3.51
TyrR	3.43
PhoP	3.32
HNS	3.32
NtrC	3.29
NagC	3.26
AgaR	3.25
GalS	3.23
IclR	3.23
Lrp	3.18
NanR	3.12
FadR	3.11
SoxS	3.03
OxyR	3.01
PhoB	2.97
EvgA	2.97
GalR	2.93
PdhR	2.92
TorR	2.73
OmpR	2.73
CsgD	2.72
FlhDC	2.71
MarA	2.7
GlpR	2.68
DgsA	2.54
GadW	2.54
GntR	2.48
MntR	2.46
GadX	2.43
CytR	2.4
TrpR	2.4
PIE (13 nt) in *ΔgreAB* strain	2.38
ExuR	2.36
Nac	2.3
PutA	2.26
Dan	2.24
AraC	2.24
SlyA	2.22
RcsB	2.18
AscG	2.18
DeoR	2.18
Rob	2.16
DcuR	2.11
NrdR	2.1
ArgP	2.1
CaiF	2.09
RcsAB	2.04

IscR	2.03
NhaR	2
FhlA	2
ModE	1.91
RstA	1.91
RutR	1.84
MelR	1.84
UxuR	1.79
HipB	1.78
MqsA	1.76
GadE	1.74
MetR	1.73
AsnC	1.69
BaeR	1.67
CysB	1.66
UlaR	1.64
RhaS	1.58
LeuO	1.54
XylR	1.5
RelBRelE	1.46
MqsAMqsR	1.45
Ada	1.4
MlrA	1.33
GcvA	1.33
