# Supplementary material for: Visualizing translocation dynamics and nascent transcript errors in paused RNA polymerases in vivo
Source: Genome Biol. 2015 May 15;16(1):98. doi: 10.1186/s13059-015-0666-5 (PMC4457086; doi:10.1186/s13059-015-0666-5)
Supplement: Additional file 3: — Method S1. Calculation of MAP scores for PIEs. Method S2. Inactivation of greA and greB genes. [file 13059_2015_666_MOESM3_ESM.pdf]

Supplementary Method 1: Calculation of the MAP scores.

The maximum a posteriori probability (MAP) score for the PIE was calculated using a sequence group collected by the parameters *P*(0.9, 100) for WT cells or *P*(0.9, 160) for *ΔgreAB* cells, respectively, according to Hertz and Stormo [1] (also see main text for the parameters). Likewise, the MAP scores for *E. coli* transcription factors were calculated using data downloaded from RegulonDB (http://regulondb.ccg.unam.mx/menu/download/datasets/files/PSSMSet.txt). For example, in the MAP score regarding the PIE for WT cells, we used 11-nt sequences of 758 positions and computed as follows:

$$MAPscore = \frac{\log_2(758)}{11} \times \sum_{i=1}^{11} \sum_{x=\{A,C,G,T\}} f_i(x) \times w_i(x),$$

$$w_i(x) = \log_2 \frac{f_i(x)}{p(x)},$$

$$f_i(x) = \frac{n_i(x) + p(x)}{758 + \sum p(x)},$$

where $f_i(x)$ is the relative frequency of a residue x at a position i, $p(x)$ is the background probability for a residue x, i.e. GC contents in *E. coli* genomic sequence (NC_007779.1) , and $\sum p(x)$ = 1 is set as pseudocount.

Reference

1. Hertz GZ, Stormo GD: **Identifying DNA and protein patterns with statistically significant alignments of multiple sequences.** *Bioinformatics* 1999, **15:**563-577.

Supplementary Method 2: Inactivation of *greA* and *greB* genes.

The *E. coli greA* and *greB* genes were inactivated by recombineering using the *tet* and *amp* drug cassettes, respectively. Recombineering is based on the λ *red*-dependent recombination of DNA substrates into the chromosomes or plasmids of enteric bacteria using short 40–50nt flanking homologies [1]. Briefly, *E. coli* DY330 cells were induced for Red functions at OD600 ~ 0.5 and prepared for electroporation with 300 ng of appropriate linear DNA cassette. The cells were recovered in 0.9 ml LB, grown for 2–3 h at 32ºC, spread on LB agar plates supplemented with 25 μg/ml Ampicilline or 12.5 μg/ml Tetracycline and incubated for 1–3 days at 32–34ºC to reveal drug-resistant recombinants. The *greA-tet* and *greB-amp* cassettes for gene disruptions were made by PCR amplification of the pBR322 *amp* and Tn10 *tetA orfs*, respectively, using Platinum Taq DNA Polymerase High Fidelity Kit (Invitrogen) with primers that contained a 39–40nt sequence homologous to chromosomal regions surrounding the gene *orf* targeted for disruption followed by a 22–25nt priming sequence complementary to the 5' and 3' regions of the drug *orf*. The resulting cassettes should have a drug *orf* flanked by the 39–40nt regions of homology that are needed for its recombination into chromosomal regions of the respective gene, so that the chromosomal *orf* is replaced with the drug *orf*.

For *greA<>tet* replacement, the *tet* cassette was made using a pair of forward and reverse primers 5’TGGGTGAAGACTTGCCCTATCAGGAATATTCAAGAGGTATAACAAATGAATAGTTCGACAAAGATCGC3’ and 5’TTCCTTTCTTTACAATACATCAACATCTTGAGTATTGGGTAATTCCTAAGCACTTGTCTCCTGTTTAC3’. For *greB<>amp* replacement, the *amp* cassette was made using a pair of forward and reverse primers 5’ATACCCAATGTGCGCATTATCAAACAGACAAAGGGAATCAACGAGATGAGTATTCAACATTTCCGTGTC3’ and 5’GGCTGGCAAAAATGCCAGCCATCGGCAGGAGGTTAAGACTCTTCCTTACCAATGCTTAATCAGTGAGGC3’.

Analysis of gene replacements.

Recombinant colonies were purified on LB plates containing appropriate drug at 34ºC. The individual colonies from the streaks were suspended in 30μl of sterile water and 1 μl used as a template for PCR with two check primers flanking the target gene.

The *greA<>tet* replacement was verified with a pair of forward and reverse primers TCTGGTCCCGGTAAGGAGTTATGC and GCAAAATGCCACGCTCTGTTCGTTG. The *greB<>amp* replacement was verified with a pair of forward and reverse primers CGGCGAAACATTATTGATTCTGTTG and AAATCAGGGGATAGTTATACGGAC.

Construction of *greAB* double mutant.

The *greAB* double mutant was constructed in the NB854 strain containing the chromosomally located 6xHis-tagged *rpoC*. The *rpoC* gene was tagged using recombineering by inserting the 6xHis-*kan* cassette at the 3' end of *rpoC* and selecting for Kanamycin-resistant colonies followed by DNA sequencing to verify the final chromosomal *rpoC*-His construct (Nina Costantino, personal communication). To construct the *greAB* mutant, *greA<>tet* and *greB<>amp* were sequentially transduced into the NB854 cells using the P1 transduction procedure and selecting for Tetracycline- and Ampicilline-resistant colonies, respectively, on LB plates at 37ºC. The resulting NB959 strain was Tetracycline- and Ampicilline-resistant with temperature-sensitive phenotype at 42ºC.

References.

1. Sawitzke JA, Thomason LC, Bubunenko M, Li X, Costantino N, Court DL: **Recombineering: using drug cassettes to knock out genes in vivo.** *Methods in enzymology* 2013, **533:**79-102.
